# Long-lead Prediction of ENSO Modoki Index using Machine Learning algorithms

**DOI:** 10.1038/s41598-019-57183-3

**Published:** 2020-01-15

**Authors:** Manali Pal, Rajib Maity, J. V. Ratnam, Masami Nonaka, Swadhin K. Behera

**Affiliations:** 1 Department of Civil Engineering, Indian Institute of Technology Kharagpur, Kharagpur, 721302 West Bengal India; 2 Application Laboratory, Japan Agency for Marine-Earth Science and Technology, Yokohama, Japan

**Keywords:** Atmospheric dynamics, Ocean sciences

## Abstract

The focus of this study is to evaluate the efficacy of Machine Learning (ML) algorithms in the long-lead prediction of El Niño (La Niña) Modoki (ENSO Modoki) index (EMI). We evaluated two widely used non-linear ML algorithms namely Support Vector Regression (SVR) and Random Forest (RF) to forecast the EMI at various lead times, viz. 6, 12, 18 and 24 months. The predictors for the EMI are identified using Kendall’s tau correlation coefficient between the monthly EMI index and the monthly anomalies of the slowly varying climate variables such as sea surface temperature (SST), sea surface height (SSH) and soil moisture content (SMC). The importance of each of the predictors is evaluated using the Supervised Principal Component Analysis (SPCA). The results indicate both SVR and RF to be capable of forecasting the phase of the EMI realistically at both 6-months and 12-months lead times though the amplitude of the EMI is underestimated for the strong events. The analysis also indicates the SVR to perform better than the RF method in forecasting the EMI.

## Introduction

The El Niño (La Niña) Modoki (ENSO Modoki, hereafter EM)^1^ is a newly acknowledged phenomenon characterized by warm (cool) central Pacific sea surface temperature (SST) flanked by cool (warm) eastern and western Pacific SSTs. The EM events affect the global climate at various time scales. The EM affects the equatorial or near equatorial countries by the modified Walker circulation with rising (sinking) motion in the central equatorial Pacific and sinking (rising) motion over the west and east Pacific during EM warm (cold) events and other parts of the globe are affected by the atmospheric teleconnections due to the distribution of heating associated with the equatorial SST anomalies during the EM events^2–8^.

Although impacts of EM events have been well established, the EM is apparently not so well predicted at long lead times by current operational climate forecast models^2,9–14^. The Bureau of Meteorology Predictive Ocean Atmosphere Model for Australia (POAMA) coupled seasonal forecast model showed a partial success in predicting differences between Modoki and canonical El Niños one season ahead with correlation coefficient more than 0.6^15,16^. APEC Climate Center (APCC) Multi-Model Ensemble (MME) seasonal forecast system shows the ability to predict the patterns of tropical Pacific SST anomaly (SSTA) of the Modoki events four months ahead with a high correlation coefficient i.e. 0.8^17^. However, the predictability of anomalous SST patterns in the APCC MME is seasonally dependent. The IAP-DecPreS near-term climate prediction system, though could predict the EMI with a good skill (correlations coefficient of 0.62 and 0.53) at 4 and 7 months lead, has limited skill (correlation coefficient 0.43) at a lead time of 10 months and beyond^18^. All the above models have difficulties in forecasting the amplitude of the EMI events though they forecast the phase of the EMI realistically. The limited skill in predicting the ENSO Modoki index (EMI) in terms of long lead times by the current seasonal forecasting systems, on the face of huge benefit in predicting it, motivated us to look for alternative methods to forecast the EMI.

Statistical EM prediction based on the non-linear Machine Learning (ML) algorithms could be a potential alternative to the dynamical model based prediction. The ML based prediction has generally shown good skill in forecasting events, though the method has limited capability to understand the underlying processes^19^. The skill of the ML algorithms stems from the use of observed data for the training. The ML algorithms, unlike climate models, are less computationally intensive. Two widely used ML algorithms namely Support Vector Regression (SVR), and Random Forest (RF) are used here.

The RF technique proposed by Breiman (2001)^20^ is popular for classification, prediction, studying variable importance, variable selection, and outlier detection. It consists of an ensemble of simple tree predictors where each tree yields a response presented with a set of predictor values. In regression problems, the responses are averaged to estimate the dependent variable. The SVR^21^ minimizes the expected error of a learning machine thus reducing the problem of overfitting. This is a robust and proficient technique for both classification and regression. A lot of studies on the applications of RF and SVR can be found where these techniques outperformed or performed with same skill as other established techniques. The studies showing the skills of these two algorithms include a study on drought forecast to predict the time series of monthly standardized precipitation index (SPI)^22^, the prediction of onset of Australian winter rainfall by RF^23^, application of RF to daily and monthly rainfall forecasting^24^, hourly rainfall forecasting by SVR^25^, reservoir inflow forecasting^26–28^, streamflow/ river stage forecasting^29,30^, typhoon flood forecasting^31^, and hydrologic time series analysis^32^. Hence, the successful application of these two ML algorithms for constructing prediction models in different fields of studies encourages the idea to use the same for long-lead prediction of the EM events.

In a nutshell, the importance of long-lead prediction of EM events to understand the resulting climatic impacts and teleconnection patterns and the lack of prediction skill of existing climatic operational systems, build the motivation for the paper. Based on the motivation, the objective of the paper is to evaluate the ability of ML algorithms to provide effective long-lead prediction of EM events, the first of its kinds, using slowly varying climatic variables as predictors. The following sections provide a detailed description of the results obtained, data used and mathematical description of the models in the methodology.

## Results

### Identification of the input regions

At the outset, the slowly varying climatic variable, global monthly SSTA, sea surface height anomaly (SSHA) and soil moisture content anomaly (SMC) at 100–289 cm depth, for the period of 1982 to 2017, are selected as the predictors for EMI. Lagged correlations between the monthly observed EMI and SSTA, SSHA and SMC are determined by Kendall’s tau (*τ*) considering the lags of 6, 12, 18 and 24 months to identify the regions significantly (at 1% significance level) associated with the EMI. The statistical significance is computed at 1% significance level after a field significance analysis using two-tailed Z-test. Distribution of Kendall’s tau approximately follows normal distribution for large number of sample. The Z-test was performed to find out the statistical significance since the data size was sufficiently large (>400). The consideration of (*τ*) helps to deal with the non-linear relationship between the input and the target. The domain of interest is selected based on the (*τ*) values, which are statistically significant at 1% significance level. The identification of the significant zones indicates that there can be several numbers of identified input zones to characterize the EMI. However, multi-dimensionality can hinder the accurate interpretation of the effective information and thus dimensionality reduction is always helpful in the prediction process. We used the Supervised Principal Component Analysis (SPCA), which is one of the most effective tools for dimensionality reduction^33,34^. The SPCA utilizes the Hilbert–Schmidt Independence Criterion (HSIC) and develops the principal components based on an orthogonal transformation of the input matrix^35^. By applying SPCA on the *n*-dimensional input set, a set of principal components is obtained in the order of its association with the target variable. Thus, the first component is expected to exhibit maximum association with the target variable. The mathematical descriptions of Kendall’s tau and SPCA technique are provided later in the methodology section. In the present study, the optimal number of principal components to be considered is determined by examining the variation of the prediction performance with number of principal components considered before feeding to the ML tools.

**Figure 1:**
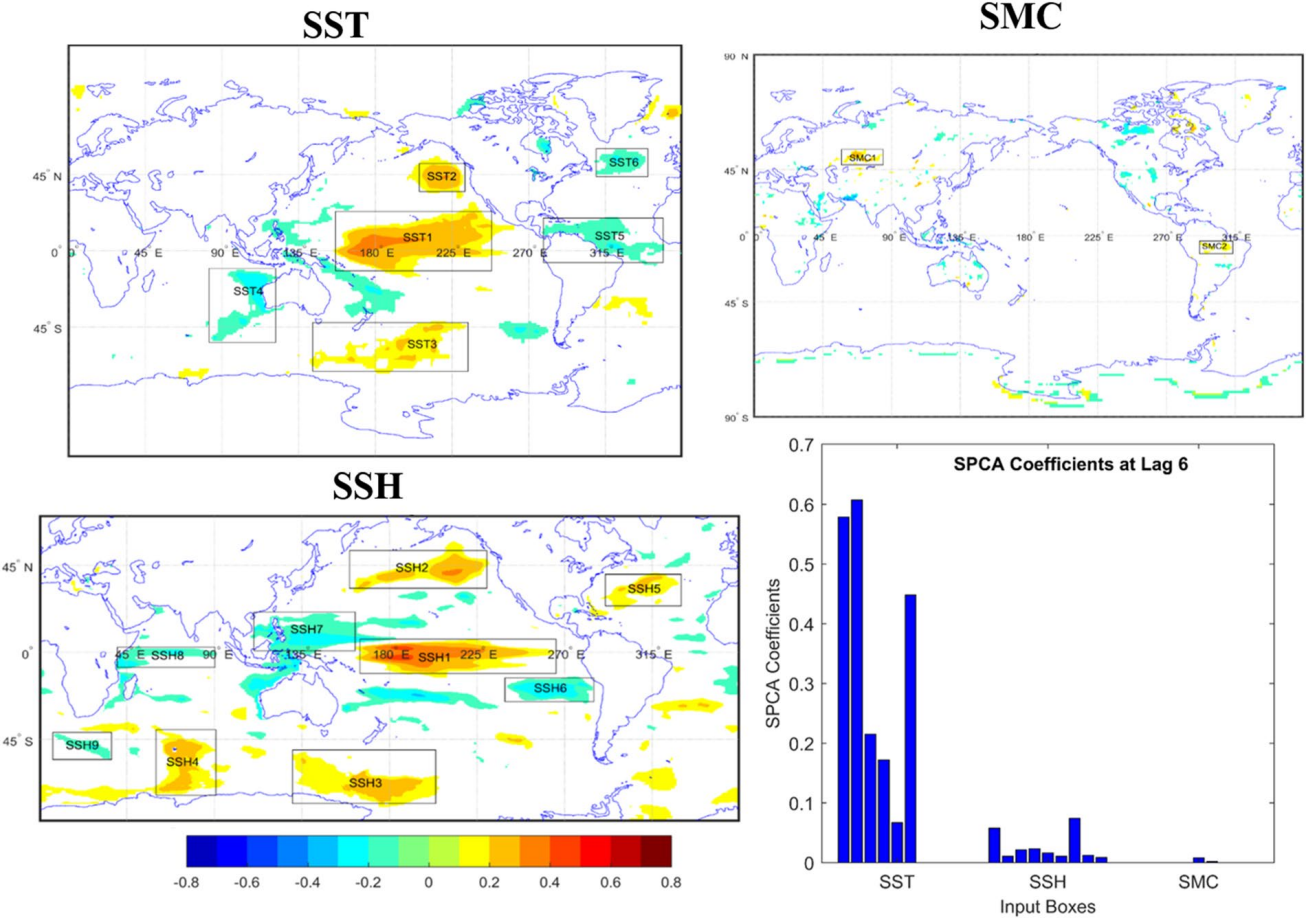
Identified significant zones from the global fields of SSTA, SSHA and SMC along with the SPCA coefficients of each identified zones at 6 months lead.

**Figure 2:**
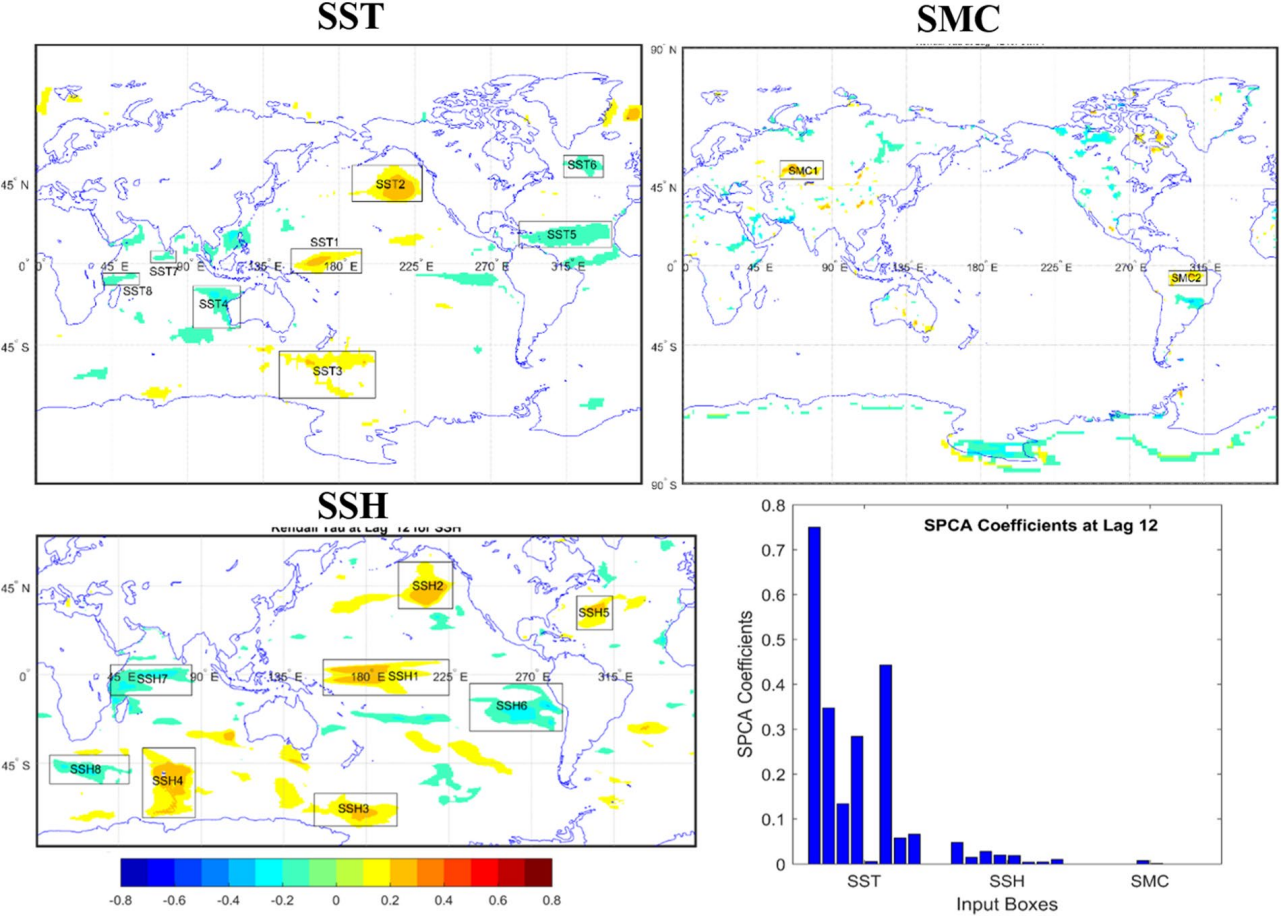
Same as Figure 1 but at 12 months lead.

Figures 1 and 2 show the identified zones of SSTA, SSHA and SMC values based on the correlation analysis and the SPCA coefficient values (the associated bar plots) of each identified regions for the leads of 6 and 12 months respectively. The same for the leads of 18 and 24 months can be found in Figs. S1 and S2 in the supplementary document. The latitude longitude information along with their monthly variance values for all the four lead times are represented in Tables S1–S4 respectively in the supplementary document. The squared values of the SPCA coefficients quantify the individual contribution of each selected zones to predict EMI for that particular lead time. A comparison among the different variables based on the values of the SPCA coefficients indicates that the SSTA fields provide maximum information to predict the EMI for all leads. The SSTA fields at Central and North Pacific region show the maximum positive correlations with the target, which is also supported by the highest SPCA coefficient values for lead 6. The contribution from the Central Pacific region decreases at higher lead time i.e. at 12, however, it still shows the maximum association with the target compared to other selected predictors. Nevertheless, the SSTA field from the same region does not show any association at all with target at the leads of 18 and 24 months. ENSO Modoki evolution in tropical Pacific basically determines the EMI relationship with SSTA. The signals from the western Pacific move to central Pacific in 6 to 12 months (e.g. Ashok *et al*. 2007). Therefore, the association in terms of correlation with EMI are not seen in central tropical Pacific at 18 to 24 months lead. On the other hand, the SSTA field over the North Pacific region is significantly correlated to EMI even at higher leads i.e. for the leads of 12, 18 and 24 months although the association is visibly decreasing (in terms of correlation) with the increase in leads. The SSTA over the North Atlantic shows significant negative correlation with the EMI at all the leads. The evolution of ENSO Modoki is associated with several tropical and extra-tropical processes (including the signals on the trails of previous ENSO and ENSO Modoki events). Some of the signals seen in the North Pacific are related to these processes. However, this study lacks the scope to discuss ENSO Modoki evolution mechanism as the focus here is only to evaluate ML approaches for long-lead prediction of EMI. The remaining identified SSTA regions are comparatively lesser contributing, however, still higher than other two climatic variables i.e. SSHA and SMC.

SSHA is found to be the second most contributing climate variable although the contribution is much lesser than SSTA. The SPCA coefficient values range from 0.003 to 0.2 for all the leads considering all the identified fields. At the lead of 6 months, the highest positive correlation of SSHA field at Central Pacific region with the EMI is explicit. Contrastingly, the SSHA field from the Western Pacific region is negatively associated with the EMI, though it has the highest contribution to the prediction among all the identified SSHA zones for lead 6. At the next lead i.e. at lead 12, the Central Pacific SSHA field shows maximum association. All the considered SSHA fields show trivial associations to predict EMI at lead of 18 months, which remain same for the lead of 24 months except the fact that the association from the Central Pacific region significantly increases perhaps owing to the resultant effect of a previous El Niño Southern Oscillation (ENSO) or EM event. Finally, considering the SMC fields, the contribution of the two identified zones in Europe and Amazon region are visibly insignificant. However, the positive correlation value (Kendall’s tau ~ 0.3–0.4) and SPCA coefficient values for the SMC field at European region remain almost same for all the leads.

As stated earlier, the study aims to minimize the multi-dimensionality problem by using the SPCA technique. It quantifies the individual contribution of each selected predictors for EMI. However, the final selection of the input fields is based on the variation of prediction performance with number of input variables considered. It has been observed that for all the leads, the prediction performances increase with the increase of number of input fields. Hence, for the present study, all the identified predictor fields except the SMC over the Amazon region, are used to develop the prediction model. The inclusion of SMC over the Amazon region does not improve the model performances at any lags and thus discarded from the set of input variables. Although, the study lacks the ability to provide any physical justification for this currently, it can be considered as a future study, as the current one emphasises on exploring the EMI predictability using ML algorithms.

### Model performance

The performances of the selected models are evaluated by different performance statistics namely Correlation Coefficient (CC), Refined Degree of Agreement (*D*_*r*_), Root Mean Square Error (RMSE) and the unbiased Root Mean Square Error (uRMSE). The models have been developed independently for all the leads i.e. 6, 12, 18 and 24 months. The independently developed models for each lead are applied and the outcomes are evaluated through performance statistics evaluated during both the development and testing periods.

**Figure 3:**
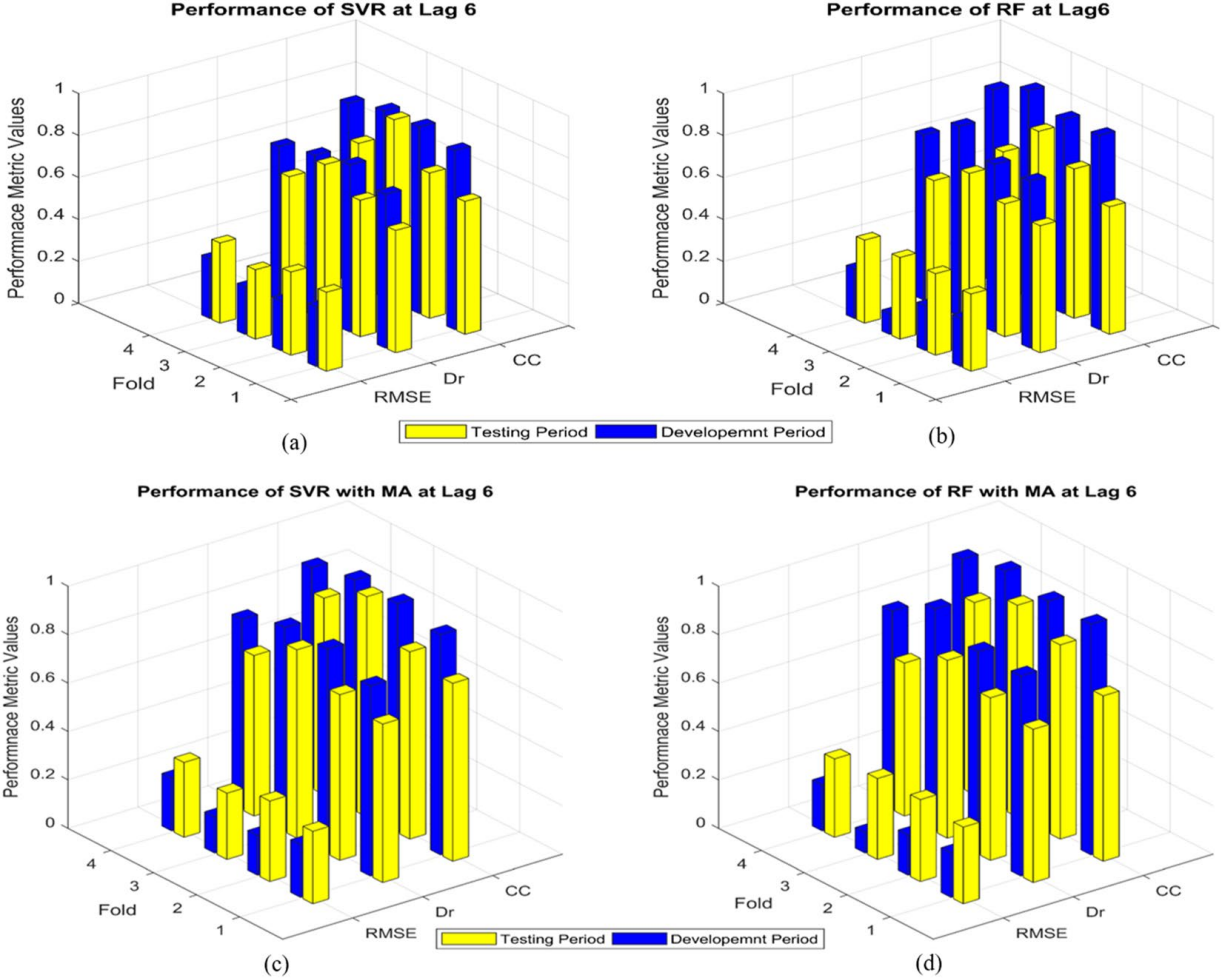
Comparison of model performances at 6 months lead time: (**a**) Performance of SVR without applying the MA; (**b**) Performance of RF without applying the MA; (**c**) Performance of SVR after applying the MA; and (**d**) Performance of RF after applying the MA.

**Figure 4:**
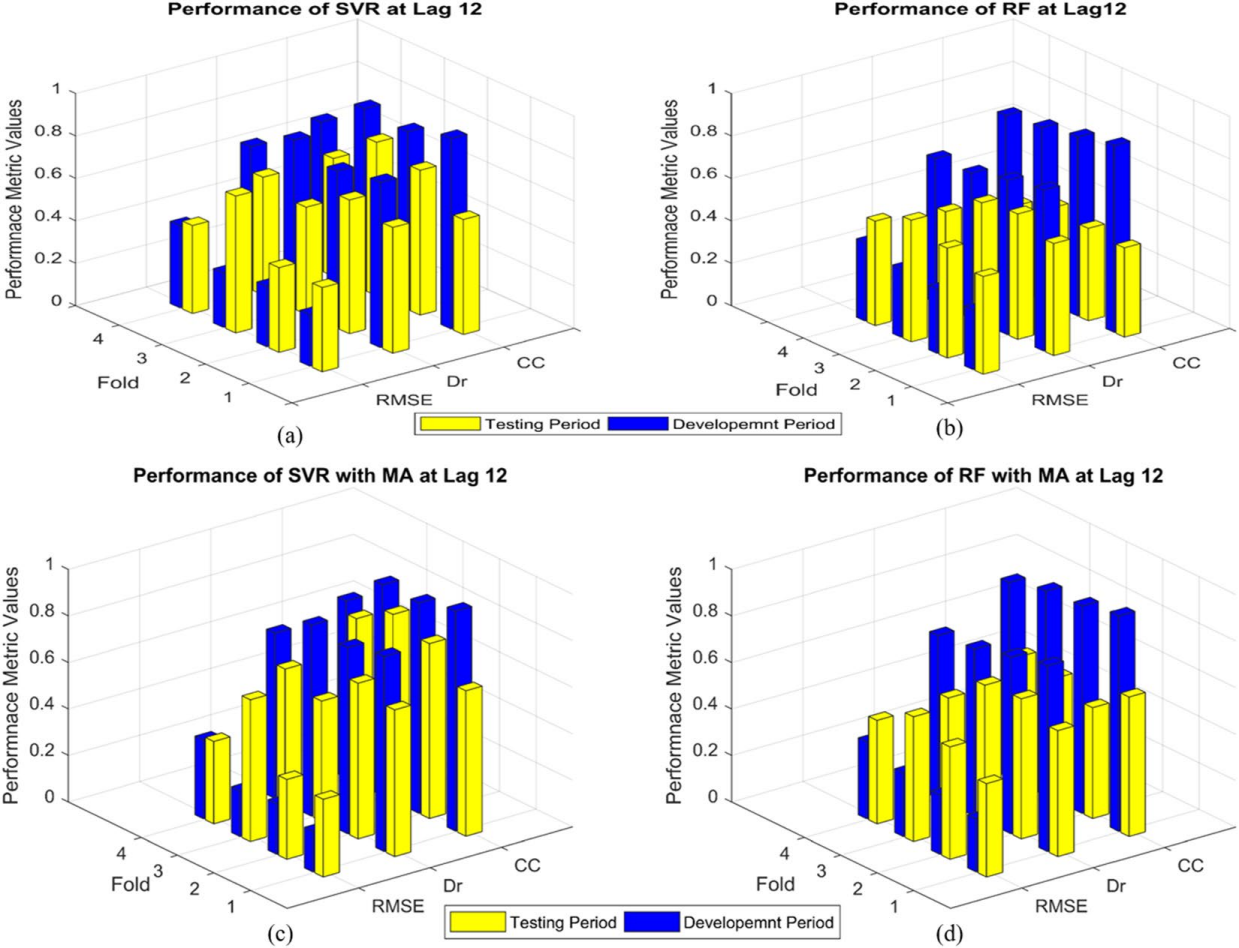
Comparison of model performances at 12 months lead time: (**a**) Performance of SVR without applying the MA; (**b**) Performance of RF without applying the MA; (**c**) Performance of SVR after applying the MA; and (**d**) Performance of RF after applying the MA.

We applied a 5-month Moving Average (MA) to the EMI to filter out the high frequency intra-seasonal variations. Figures 3 and 4 show the comparison of model performances for the SVR and RF at the lead of 6 and 12 months during model development and testing periods with and without the application of the low-pass filter (Tables S5 and S6 show the values of the model performance metrics for the two cases in the supplementary document). The application of the low-pass filter enhances the skill scores of the ML models. However, the two cases are found to exhibit similar skills in performance metrics across the folds and leads. Hence, most of the conclusions discussed in the following section hold true for both the cases except for some of the instances that are discussed especially wherever necessary. The outstanding model performances for both the SVR and RF during model development and testing periods at lead 6 are explicitly visible from the metrics values for both the cases introduced above. At the lead 12 months, the performance of RF decreases drastically during the testing period, although the model development shows an acceptable result. However, in case of SVR, although the model performance decreases both for development and testing periods at the lead of 12 months, the performances are reasonably acceptable considering such a sufficiently long lead time. The next lead i.e. 18 months, shows the worst performances for both the SVR and RF perhaps owing to the overtraining of both the models. Nevertheless, the performances for the two models improve in terms of overfitting for the lead of 24 months though the model performances are not acceptable. The performances for the two cases of with and without the short-term fluctuations for the leads of 18 and 24 months can be found in Figs. S3 and S4 in the supplementary document. Briefly, the comparison among different lead times shows that the SVR is able to skilfully predict the EMI up to a lead of 12 months. In case of RF, although the model is able to predict quite accurately at the lead of 6 months, its performance is not good at the lead of 12 months. Beyond that, i.e. for the leads of 18 and 24 months, both the models loose skills to predict owing to too long lead time to capture the variation.

**Figure 5:**
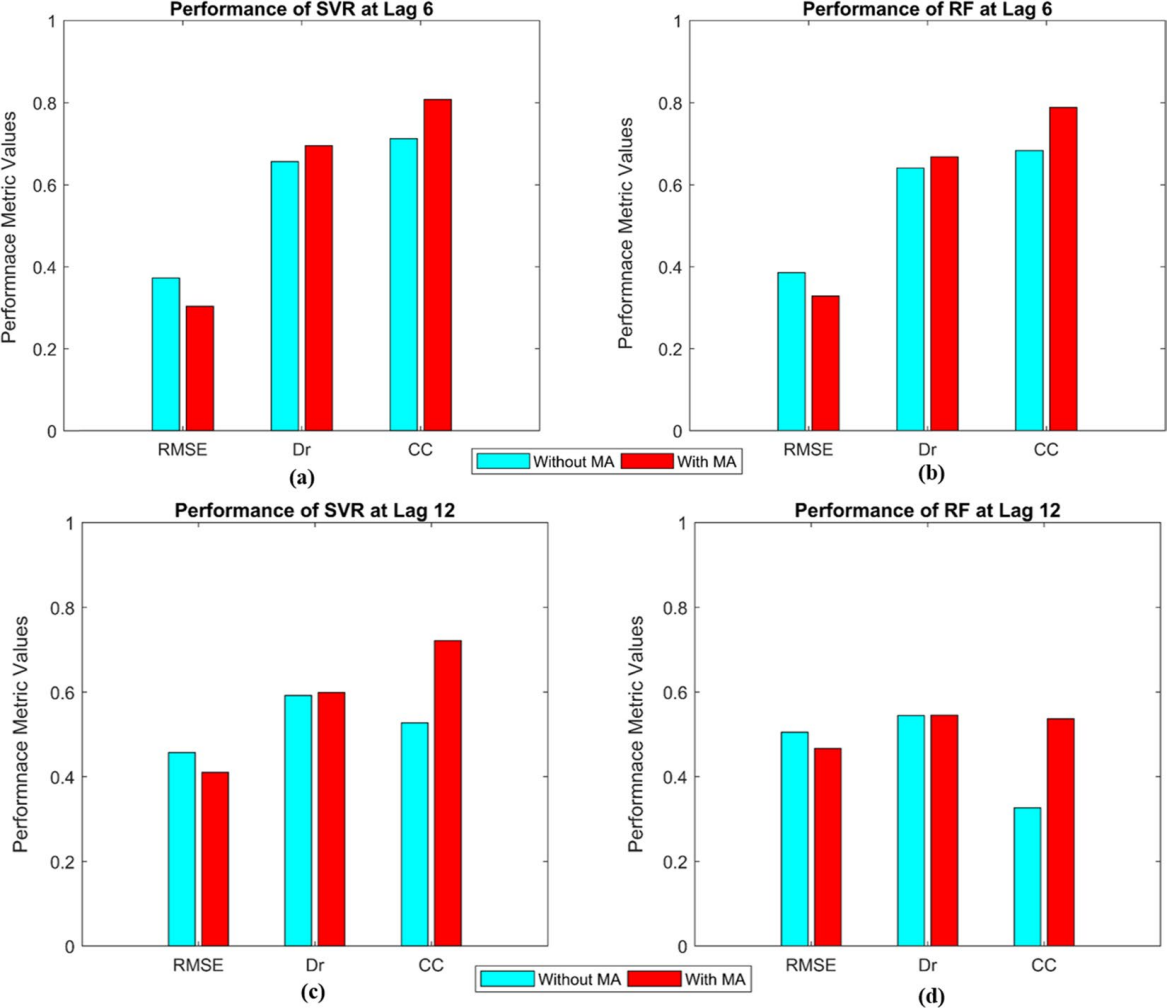
Comparison of overall model performances without and with applying the MA to remove short-term fluctuations: (**a**) Performance of SVR at lead time of 6 months; (**b**) Performance of RF at lead time of 6 months; (**c**) Performance of SVR with lead time of 12 months; and (**d**) Performance of RF with lead time of 12 months.

The overall performances of the models at all the leads are further evaluated by computing the model performance metrics considering the data from the development and testing periods of each fold as one time series. Also, the correlation coefficient value between EMI of testing period at lead 6 and that at lead 12 is computed as 0.787 (shown as average of all four folds). Figure 5 shows the overall performances for those two cases with and without the short-term fluctuations for the leads of 6 and 12 months. The same for the leads of 18 and 24 months are provided in the supplementary document in Figure S5. The same inference as discussed above is drawn from both the figures; i.e. the model performances are acceptable up to the lead time of 12 months. Additionally, the model performance is compared with the persistent of EMI, which is computed as follows: the observed EMI is used as the predicted values at the lead of *N*-months (6 or 12 months as examples) and correlated with the actual observed values of that month for the evaluation. Following this, the correlation coefficients for the leads of 6 and 12 months are found to be 0.596 and 0.271 respectively. Whereas, the correlations obtained from SVR and RF predicted EMI range between 0.735 to 0.952 and 0.683 to 0.990 respectively for the lead of 6 months in development and testing periods. The same metrics, for the lead of 12 months are 0.626 to 0.946 and 0.476 to 0.927 for SVR and RF respectively. Evidently, the correlations obtained from model predicted EMI are much higher than the persistence of correlation of EMI for the lead time of 6 and 12 months.

The comparison among the two models indicates the better performance of SVR for all the leads compared to RF. In RF, the peaks are captured very well in model development period. However, the ability to capture the amplitude of the extreme variabilities reduces in testing periods for all the folds and leads, although still able to capture the phases. The difference between the model performance during development and training periods can be reduced to some extent by tuning the *m*_*try*_ number and *nodesize* for each case. However, the tuning process was investigated and found to produce the predicted values almost equal to the mean of the observed values without capturing the variations and the extreme values. This could perhaps be due to the short length of the observed data. It may be noted that the model development criteria are maintained uniformly for both RF and SVR to capture the variation and extreme values of EMI at an acceptable level. In case of SVR, although the performance metric values are lesser than RF during the development period, there is a parity of performance between development and testing periods and it is able to capture the peaks reasonably well in the testing periods also. Considering all these issues, SVR is found to be a better option for the prediction of EMI even at higher leads. The application of these two approaches in different fields of studies and comparisons of their performances show that the advantages of these two models, while compared to each other, are very much problem dependent^36^. In our study, the comparative analysis of the performances of the two models indicates the suitability of SVR for predicting EMI at 6 and 12 months lead with short data period.

**Figure 6:**
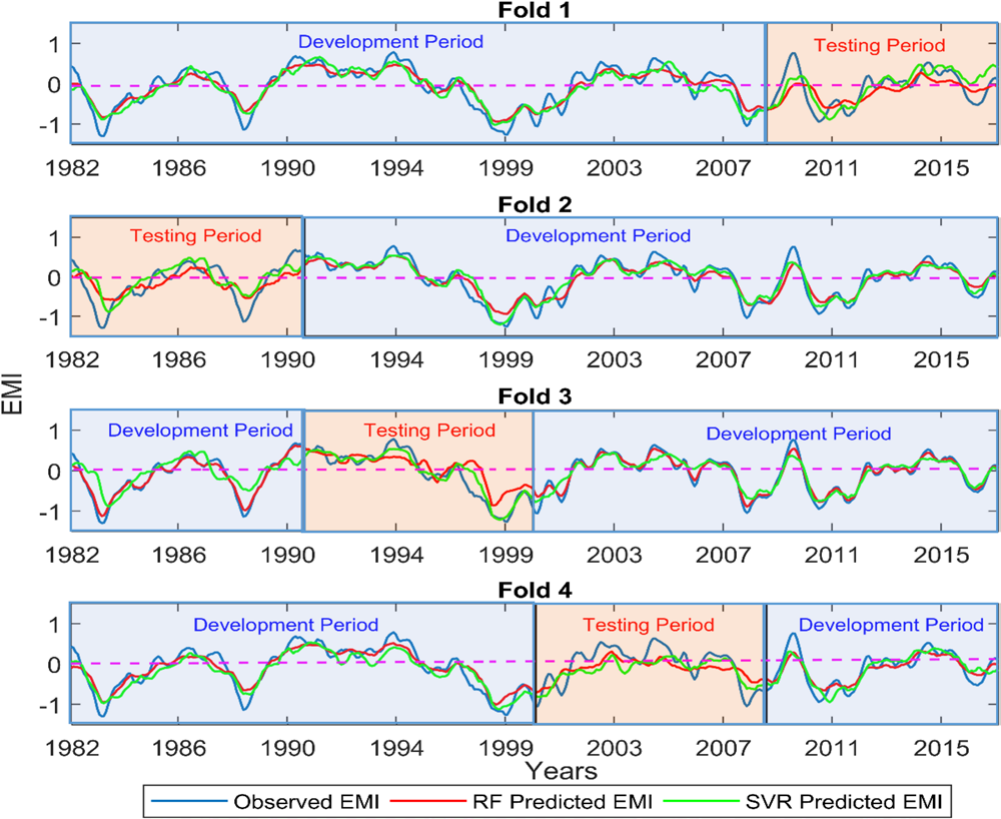
Comparison of observed EMI with the RF-predicted and SVR-predicted EMI at 6 months lead time after removing short-term fluctuations (5-month moving average).

**Figure 7:**
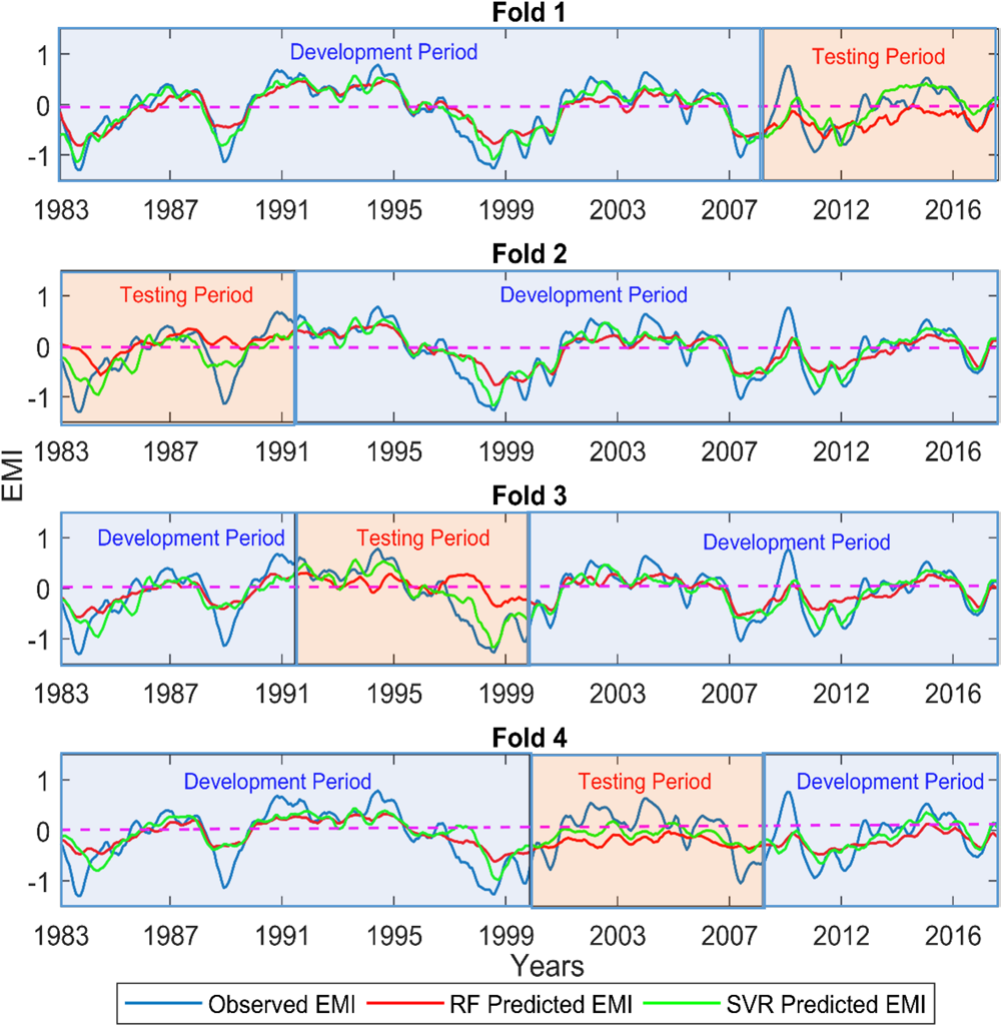
Comparison of observed EMI with the RF-predicted and SVR-predicted EMI at 12 months lead time after removing the short-term fluctuations (5-month moving average).

Figures 6 and 7 show the time series plots of observed and predicted EMI for all the models and folds for the leads of 6 and 12 months for the case of without short-term fluctuations. The counterparts i.e. the time series plots of observed and predicted EMI for all the models and folds for the leads of 6 and 12 months with short-term fluctuations are shown in Figures S6 and S7 in the supplementary document. The comparison of these two cases shows that although the model performances are improved after reducing the short-term fluctuations, the models’ abilities in terms of capturing the peaks are not improved significantly. The model performances are not uniform across the four folds. It is observed that for the first fold, where the model development period is approximately 1982 to 2009 and testing period is from 2009 to 2017, the SVR and RF perform the best, especially in testing periods. Particularly, the SVR is able to capture the EM events for both the development period (approximately the years of 1983 and 1999) and testing period (the year of 2010). The SVR-predicted EMI also shows a good association with the observed data for the frequent and shorter peaks throughout the length of the time series. On the other hand, the RF shows an overfitting tendency, i.e. it captures the Modoki events well during the model development period but fails to do so during testing period where it only captures the direction/phase of the peak. The second fold, where the EMI data from the year 1991 to 2017 (approximately) is used to develop the models, shows a similar trend to that of fold 1, although the time series plots show a little deterioration in the ability to capture the shorter as well as extreme peaks for both the SVR and RF models. The third and the fourth folds, give EMI prediction without capturing any of the peaks. At the lead of 12 months, the abilities to capture the peaks decrease drastically for both the models. However, the SVR shows a superior skill than RF to capture the higher peaks of EMI in the testing periods, particularly for the first and the second folds. Similar to the lead of 6 months, the third and fourth folds show not so good model performances for both SVR and RF at leads of 12 months. It is interesting to note that both SVR and RF models could capture the correct phases of the EMI events in all the four phases in most of the years. However, the amplitudes of the EMI are underestimated by both the models. The above discussion of different trends of model performances across the four folds are same for the cases of with and without short term fluctuations as mentioned earlier in the paper. The prediction at 18 and 24 months leads are not acceptable, yet for both the leads the model performances follow the same trend of performing better in first and second folds. This can be perceived in the comparison of observed and predicted EMI for these two leads in Figures S8–S11, provided in the supplementary document.

Next, observed Modoki events away from its mean by more than one standard deviation (STD) are specifically picked out for case by case evaluation of prediction performance by SVR. Table 1 shows such periods, observed phases of the Modoki events (positive/negative) along with the predicted phases in case of 6-month as well as 12-month lead times. The prediction ability is evaluated in following four categories:- (a) both phase and magnitude are accurately (away from mean by more than one STD) predicted and designated as (√√√); (b) phase is correctly predicted but the magnitude is predicted marginally lesser than one STD which is designated as (**√√**); (c) phase is correctly predicted but the magnitude is predicted poorly, i.e., much less than the STD value (**√**); and (d) incorrect prediction of phases and amplitudes (**×**). For the lead of 6 months, more accurate predictions of magnitudes and phases are noticed in 1983–1984, 1991–1992, 1998–1999, and 2000. In the years of 1988–1989, 1990–1991, 1994–1995, 2001, 2008–2009, and 2011–2012, predictions of correct phases are noticed but relatively poorer performances in terms of marginally underestimated magnitudes, i.e. falling in the second category of the prediction ability. The phases are predicted correctly but poor predictions of magnitudes are observed for the events occurred in the years 1998, 2006, 2009–2010, 2015 and 2016. For the lead time of 12 months, in general, the overall performance is poorer than 6-month lead time prediction as expected. However, still the ability to capture both phases and magnitude in some of the cases (1991–1992, 1994–1995 and 2011–2012) is noted. The prediction ability falls in the second category in the years of 1983–1984, 1988–1989, 1990–1991, 2008–2009, 2010–2011 and 2015 when the predicted magnitudes marginally fail to capture one STD away values but captures the exact phases of the Modoki events. In a nutshell, for both the lead times, 45% of the prediction cases fall in the first or second category i.e. almost correctly predict both the amplitudes and phases. The rest of the identified time periods fall either in third (**√**) or fourth (**×**) category of prediction, which may be understandable, considering the long lead times of 12 months.

**Table 1:** Case by case evaluation of prediction performance by SVR. The table shows the periods of Modoki events away from its mean by more than one standard deviation and corresponding observed and predicted phases (positive/negative). The prediction ability is evaluated in four categories i.e.: (a) both phase and magnitude are accurately (away from mean by more than one STD) predicted, designated as (**√√√**); (b) phase is correctly predicted but the magnitude is predicted marginally lesser than one STD, designated as (**√√**); (c) phase is correctly predicted but the magnitude is predicted poorly, i.e., much less than the STD value (**√**); and (d) incorrect prediction of phase and amplitude (**×**).

Periods	Phase	Prediction Lead Time 6-month 12-month	
April, 1983–April, 1984	Negative	√√√	√√
October, 1988–June, 1989	Negative	√√	√√
November, 1990–June, 1991	Positive	√√	√√
October, 1991–January, 1992	Positive	√√√	√√√
August, 1994–June, 1995	Positive	√√	√√√
October, 1997–December, 1997	Negative	×	×
January, 1998–May, 1998	Negative	√	×
June, 1998–September, 1999	Negative	√√√	√
January, 2000–June, 2000	Negative	√√√	√
January, 2001–May, 2001	Negative	√√	√
August, 2002–October, 2002	Positive	×	×
August, 2004–December, 2004	Positive	×	×
March, 2006–April, 2006	Negative	√	×
January, 2008–March, 2009	Negative	√√	√√
November, 2009–March, 2010	Positive	√	×
September, 2010–June, 2011	Negative	√	√√
December, 2011–June, 2012	Negative	√	√√√
March, 2015–April, 2015	Positive	√	√√
November 2016	Negative	√	√
March, 2017–April, 2017	Negative	×	√

Thus, the advantage of prediction using ML approach to determine the correct phases of the Modoki events with long lead-times (6 and 12 months) is creditable as compared to the existing climate forecast models as discussed before in Section 1. Moreover, the predictions of amplitudes, specifically from SVR, are notably advantageous in most of the cases with 6-month lead time, whereas the existing prediction models are strictly limited to distinguish the patterns 0–2 months ahead.

## Conclusions

Long-lead (6 to 12 months) prediction of EMI with reasonable accuracy is achieved in this study using two ML algorithms namely SVR and RF. The input to the models were the anomalies of the slowly varying climatic variables such as SSTA, SSHA and SMC. Firstly, the correlation analysis with Kendall’s tau helps to identify the significantly contributing signals from global anomaly fields of each predictor considering the non-linear dynamics. Subsequently, the study uses the SPCA technique to reduce the dimensionality which also ensures the selection of predictors having maximum association with the target i.e. the EMI. The SPCA analysis shows the coefficients corresponding to SSTA fields have the highest contributions in EMI predictions as compared to that of SSHA and SMC. SSTA from the Central and Northern Pacific regions along with the signal from Northern Atlantic region are having the maximum association with the EMI. While identifying, along with the already established fields connected with the EMI such as SSTA and SSHA from central pacific region, some additional fields are identified. These additional fields such as, SSTA signals from Northern Pacific region and Northern Atlantic region, SSHA signal form Indian Ocean region and SMC signal from Europe, are found to have significant correlation even at the higher leads of 12, 18 and 24 months. However, enhancement of model performances allows the study to include almost all the SSTA, SSHA and SMC fields despite having low SPCA coefficients. The predictor selection leads to the development of SVR and RF prediction models at the leads of 6, 12, 18 and 24 months.

The results reveal that the SVR gives consistently better performance for all the leads as RF exhibits a tendency to overfitting. Regarding the leads, both the SVR and RF show excellent performances for the 6-month ahead prediction. The predictability decreases for 12 months lead prediction for both the models though the phases of Modoki events are still captured properly. Yet, the model performance of SVR is reasonably well considering the leads whereas the RF consistently shows the problem of overfitting. However, the performance of RF has improved at the lead of 12 months by reducing the short-term fluctuations. The skills of 18 and 24 months lead predictions are not acceptable and thus the idea of developing prediction models at those leads is discarded. Overall, the study concludes that a skillful long-lead prediction, i.e. 6 to 12 months ahead prediction, of El Niño Modoki events is possible with the ML algorithms such as SVR using the SSTA, SSHA and SMC fields as the predictors.

### Data

The following data sets for the period 1982–2017, are used in this study: a) monthly Sea Surface Temperature Anomaly (SSTA) data from Optimum Interpolation Sea Surface Temperature (OISST) from National Centers for Environmental Information (NOAA) at a spatial resolution of 1.0° × 1.0°; (b) monthly Sea Surface Height Anomaly (SSHA) data from NCEP Global Ocean Data Assimilation System (GODAS) at a spatial resolution of 0.5° × 0.5° and its accuracy is established by several previous studies^37–39^; and c) monthly Soil Moisture Anomaly (SMC) data at 100–289 cm depth from ERA Interim Data from European Centre for Medium Range Weather Forecast (ECMWF) at a spatial resolution of 0.75° × 0.75°. The accuracy and quality of the SMC product has been investigated in several studies based on the ground observations in recent past for surface as well as root zone depths^40–42^.

The target variable i.e. the EMI is available and thus obtained from the website of Japan Agency for Marine-Earth Science and Technology (JAMSTEC; http://www.jamstec.go.jp/aplinfo/sintexf/DATA/emi.monthly.txt). The EMI can be represented by the following equation,

1 $${\rm{EMI}}=[{\rm{SSTA}}]{\rm{A}}-0.5\times [{\rm{SSTA}}]{\rm{B}}-0.5\times [{\rm{SSTA}}]{\rm{C}}$$

The Eq. (1) represents the area-averaged SSTA over each of the region A(bounded by 165°E–140°W, 10°S–10°N), B (bounded by 110°W–70°W, 15°S–5°N), and C (bounded by 125°E–145°E, 10°S–20°N), respectively.

## Methodology

The overall methodology consists of mainly two steps. The first step is to identify and select the regions for each input variable i.e. the SSTA, SSHA and SMC which are highly associated with the target variable EMI. The initial selection of associated input regions is based on lagged correlation analysis using Kendall’s tau at the specified leads of 6, 12, 18 and 24 months at the significance level of 0.01. The areas of significant correlations are investigated for each fold and each lag individually (however, not shown to avoid redundancy), and the most common areas identified for all the folds are selected as the final contributing areas for the predictors. Subsequently, the study attempts to deal with multi-dimensionality problem using the Supervised Principal Component Analysis (SPCA). Although, as discussed above, the final selection of the contributing input zones is based on the model performances. After that, the final step of the study is the prediction model development considering the selected input variable zones and comparison of the model performances at the leads of 6, 12, 18 and 24 months using two ML approaches i.e. the Support Vector Regression (SVR) and Random Forest (RF). The mathematical descriptions of all the steps are elaborated in the following sections.

### Predictor selection based on Kendall’s Tau and Supervised Principal Component Analysis

The associated zones of the SSTA, SSHA and SMC are identified using the Kendall’s Tau (*τ*) as discussed above. It is a rank-based, non-parametric statistical measure which is defined by the difference between the probability of concordance and discordance of two random variables^43^. Suppose, *V* and *Y* are the input variable and EMI respectively. Mathematically, Kendall’s Tau can be represented by following equation:

2 $$\tau =P[({V}_{i}-{V}_{j})\,({Y}_{i}-{Y}_{j}) > 0]-P[({V}_{i}-{V}_{j})\,({Y}_{i}-{Y}_{j}) < 0]$$

where, *i* and *j* are any two time steps which are not equal $(i.\,e.i\ne j)$.

After identifying the statistically significant associated zones at 1% significance level, the study intends to diminish the redundancy of information due to multi-dimensionality using Supervised Principal Component Analysis (SPCA) performed on the development period dataset. Let, a set of *n* observed data points during development period each comprising of *p* characteristics form a matrix, *X* of *p* × *n* dimension and *Y* is the *1 × n* dimensional matrix of the output variable. The SPCA technique aims to find the subspace ${U}^{T}X$ to maximize the association between the output variable *Y* and the projected input matrix *U*^*T*^ *X* using HSIC, where *U* is an orthogonal projection matrix of size *p* × 1. The orthogonal transformation matrix, *U* which maps the data points to a space where features are not correlated, is solved by the following optimizing problem,

3 $$\begin{array}{r}{\rm{\arg }}\,{\rm{\max }}\\ U\end{array}tr({U}^{T}XHLH{X}^{T}U),subject\,to:U{U}^{T}=1$$

where, the $$\begin{array}{r}{\rm{\arg }}\,{\rm{\max }}\\ U\end{array}$$ indicates a maximization problem considering *U* as an argument. The symmetric and real matrix $Q=XHLH{X}^{T}$ of size *P* × *P*, has *P* number of eigenvalues (${\lambda }_{1}\le \mathrm{..}.\le {\lambda }_{p}$) and corresponding eigenvectors $[{\nu }_{1},\mathrm{..}.,{\nu }_{p}]$, each consisting of *P* number of elements. Generally, maximum value of the cost function is ${\lambda }_{p}+{\lambda }_{p-1}+\mathrm{..}.+{\lambda }_{p-d+1}$ and the optimum solution is $U=[{\nu }_{p},{\nu }_{p-1},\mathrm{..}.,{\nu }_{p-d+1}]$, where *d* is the dimension of $[{U}^{T}X]$. Hence, $U=[{\nu }_{p}]$, which produces the coefficients for *P* different input variables and ensures the best association of the product to the output variable. Physically, the coefficients provide information on the weightages for each of the considered input variables. The square of the SPCA coefficients represent the contribution of each input variable to estimate the target output and the sum of the squares of the SPCA coefficients is equal to one. Therefore, the comparison of the absolute values of the SPCA coefficients corresponding to each of the specific input helps to select the best possible combination of inputs for EMI prediction. Additionally, it ensures the selected combination of the input variables have the maximum association with the target variable.

### Support Vector Regression (SVR)

Support Vector Regression (SVR) has been popular in many disciplines nowadays which uses a penalty term added to the error function to penalize the resultant complexity. It aims to decrease dthe problem of overfitting by adopting the theory of structural risk minimization. The current study uses the SVR for constructing the EMI prediction models at the leads of i.e. 6, 12, 18 and 24 months. A brief mathematical description of SVR is as follows.

Let, $[({x}_{1},y{}_{1}),({x}_{2},{y}_{2}),\ldots ,({x}_{i},{y}_{i}),\ldots ({x}_{l},{y}_{l})]$ be a training dataset where *x*_*i*_ is an input vector with its corresponding output vector *y*_*i*_ and *l* is the number of data pairs. The SVR finds a regression function $f(x)=\langle w,x\rangle +b$ to represent the dependency that best describes the observed output *y* with an error tolerance *ε*, where *w* and *b* are the weighting vector and bias respectively. For this purpose, the original input domain is mapped onto a higher dimensionality space, where the function underlying the data is assumed to be linear. The SVR problem in the transformed space is identified by solving the following optimization problem,

4 $$\begin{array}{ll}Minimize & \frac{1}{2}{\Vert w\Vert }^{2}+C\mathop{\sum }\limits_{i=1}^{L}({\xi }_{i}\,+\,{\xi }_{i}^{\ast })\\ Subject\,to & \{\begin{array}{l}{Y}_{i}-\mathop{\sum }\limits_{j=1}^{K}\mathop{\sum }\limits_{i=1}^{L}{w}_{j}{x}_{ji}-b\le \varepsilon +{\xi }_{i},\\ \mathop{\sum }\limits_{j=1}^{K}\mathop{\sum }\limits_{i=1}^{L}{w}_{j}{x}_{ji}-{y}_{i}\le \varepsilon +{\xi }_{i}^{\ast },\\ {\xi }_{i},{\xi }_{i}^{\ast }\ge 0,\end{array}\end{array}$$

where, *ε* is the Vapniks insensitive loss function when data are outside of the tube of error tolerance; *C* is the capacity parameter cost which is a positive constant that determines the degree of penalized loss when a training error occurs to tune the trade-off between model complexity and tolerance to empirical errors; and ${\xi }_{i}$ and ${\xi }_{i}^{\ast }$ are called the slack variables which measure the distance (in the target space) of the training samples lying outside the *ε*-insensitive tube from the tube itself^44^. The functional dependency *f(x)* can be written as,

5 $$f(x)=\mathop{\sum }\limits_{j-1}^{K}{w}_{j}{x}_{j}+b$$

where, *K* is the number of support vectors.

The optimization problem is solved using the dual formulation subject to constraints in the loss function and introducing the Lagrange multipliers, ${\alpha }_{i}$ and ${\alpha }_{i}^{\ast }$. By solving the optimization problem the final prediction function is:

6 $$f^{\prime} (x)=\sum _{i\in N}({\alpha }_{i}-{\alpha }_{i}^{\ast })k({x}_{i},x)+b$$

where, $k(\ldots )$ is kernel function which computes non-linear dependence between the two input variables *x*_*i*_ and *x* where *x*_*i*_ are the “support vectors” and *b* is the bias. In the present study, the Radial Basis Function (RBF) kernel is used in the prediction of EMI. It is proven the best among several possibilities for the choice of kernel function, including linear, polynomial, sigmoid and splines, because of its excellent performance in capturing nonlinear relationship^45,46^. Mathematically, the RBF with kernel width − γ, can be represented as,

7 $$k({x}_{i},x)=\exp \,(\,-\gamma {\Vert x-{x}_{i}\Vert }^{2},\,\,\,\gamma  > 0$$

### Random forest (RF)

RF is one of another ML algorithm for predictive analytics consisting of an ensemble of simple trees. The two major components of RF algorithm are: (1) randomness and (2) ensemble learning.

1. *Randomness*

- *n*_*tree*_ bootstrap samples are randomly selected from the data set of size N with M features with replacement. For each bootstrap, approximately two thirds of the entire dataset is chosen as a subset (i.e. around one-third of the subset are replicated in the subset) to develop the decision tree model. The un-chosen one-third samples in the original dataset are called out-of-bag (OOB) data. This OOB data is used to get unbiased estimates of the regression error and the importance of the variables used for constructing the tree.
- For each of the bootstrap samples a regression tree is grown as such that at each node, a subset of the predictor variables (*m*_*try*_ < M) is randomly selected to generate the binary rule to make the decision for the best split. The predictor with the lowest residual sum of squares is selected for the split. Tuning of this parameter is needed for optimal performance although it is not very sensitive to the model performance.

2. *Ensemble learning*

- A subset of size N’ (bootstrap sample) with *m*_*try*_ features is drawn after random selection process to construct a single decision tree to the largest extent possible without pruning for each of the *n*_*tree*_ tree.
- Finally, predictions are calculated as all the *n*_*tree*_ trees vote upon the observation of the test data set or the OOB observation. In this ensemble learning method each of the decision trees inside the ensemble contributes individually. The final estimate is obtained by averaging the results from individual trees^20^.

### Model tuning

For all ML algorithms, *k*-fold cross-validation method is performed to determine the optimal model settings and evaluating the generalized model performance to an independent data set^47,48^. The *k*-fold cross validation also helps to avoid overfitting. To apply the *k*-fold cross validation, the dataset is randomly partitioned into 4 equally sized folds for the present study. Thus, every fold is a subset (1/4) of the complete time series. Models were then fitted by repeatedly leaving out one of the folds. The models are tuned individually for all the folds for all the four leads. A model’s performance is determined by predicting on the fold left out.

The RF implementation of the “randomForest” package^49^ in R was applied. The number of predictor variables randomly selected at each split (*m*_*try*_) was tuned for each value between one and the number of input variables^19^. The number of trees (*n*_*tree*_) was set to 500 after no increase of accuracy was observed after 500 trees. The “e1071” package^50^ in R provided the SVR algorithm used in this study. The cost, gamma and *ε*-insensitive loss function values were tuned for 2 to 512; 0.001 to 1 and 0.001 to 1 respectively. A radial kernel function was used to account for non-linearity. Table 2 shows the values of SVR and RF parameters for all the folds and leads considered in the study.

**Table 2:** Tuning parameters of SVR and RF Model for different folds and different leads.

Parameters	Lead 6					Lead 12				Lead 18				Lead 24			
	Fold					Fold				Fold				Fold			
	1	2		3	4	1	2	3	4	1	2	3	4	1	2	3	4
**Support Vector Regression Parameters**																	
Cost (C)	50		5	20	20	10	18	5	5	1	4	4	4	10	10	25	10
Kernel Width (γ)	0.01		0.01	0.01	0.005	0.01	0.005	0.009	0.001	0.05	0.009	0.002	0.001	0.001	0.001	0.1	0.01
Insensitive parameter (ε)	0.9		0.1	0.5	0.9	0.4	0.4	0.01	0.009	0.01	0.1	0.01	0.09	0.001	0.09	0.1	0.09
**Random Forest Parameters**																	
*n*_*tree*_	500	500		500	500	500	500	500	500	500	500	500	500	500	500	500	500
*m*_*try*_	5	13		10	13	13	10	19	3	1	10	5	3	10	10	5	10
*nodesize*	40	40		3	50	56	66	98	100	10	20	50	40	60	40	50	80

## Supplementary information

Supplementary Document.
