# Supplementary material for: Long-lead Prediction of ENSO Modoki Index using Machine Learning algorithms
Source: Sci Rep. 2020 Jan 15;10:365. doi: 10.1038/s41598-019-57183-3 (PMC6962158; doi:10.1038/s41598-019-57183-3)
Supplement: Supplementary file 1 — Supplementary Document. [file 41598_2019_57183_MOESM1_ESM.docx]

**Supplementary Document**

**Long-lead Prediction of ENSO Modoki Index using Machine Learning algorithms**

Manali Pal^1^, Rajib Maity1^[[1]](#footnote-1)^, J.V. Ratnam^2^, Masami Nonaka^2^ and Swadhin K. Behera^2^

^1^ Department of Civil Engineering, Indian Institute of Technology Kharagpur, Kharagpur – 721302, West Bengal, India

^2^Application Laboratory, Japan Agency for Marine-Earth Science and Technology, Yokohama, Japan

This document contains additional tables and figures to support the findings in the main article. List of additional tables and figures are as follows.

**List of Tables:**

Table S1: Latitude/longitude limits of the input variables along with the monthly variance for 6 months lag.

Table S2: Latitude/longitude limits of the input variables along with the monthly variance for 12 months lag.

Table S3: Latitude/longitude limits of the input variables along with the monthly variance for 18 months lag.

Table S4: Latitude/longitude limits of the input variables along with the monthly variance for 24 months lag.

Table S5: The model performance metrics for the SVR and RF at four folds and at 6, 12, 18 and 24 months lead time without the removal of short-term fluctuations.

Table S6: The model performance metrics for the SVR and RF at four folds and at 6, 12, 18 and 24 months lead time after removing the short-term fluctuations.

**List of Figures:**

Figure S1: Identified significant zones from the global anomaly fields of SSTA, SSHA and SMC along with the SPCA coefficients of each identified zones at 18 months lag.

Figure S2: Same as Figure S1 but at 24 months lag.

Figure S3: Comparison of model performances with 18 months lead time: (a) Performance of SVR without applying the MA; (b) Performance of RF without applying the MA; (c) Performance of SVR after applying the MA; and (d) Performance of RF after applying the MA.

Figure S4: Comparison of model performances with 24 months lead time: (a) Performance of SVR without applying the MA; (b) Performance of RF without applying the MA; (c) Performance of SVR after applying the MA; and (d) Performance of RF after applying the MA.

Figure S5: Comparison of overall model performances without and with applying the MA to remove short-term fluctuations: (a) Performance of SVR with lead time of 18 months; (b) Performance of RF with lead time of 18 months; (c) Performance of SVR with lead time of 24 months; and (d) Performance of RF with lead time of 24 months.

Figure S6: Comparison of observed EMI with the RF-predicted and SVR-predicted EMI with 6 months lead time without the removal of short-term fluctuations.

Figure S7: Comparison of observed EMI with the RF-predicted and SVR-predicted EMI at 12 months lead time without the removal of short-term fluctuations.

Figure S8: The comparison of observed EMI with the RF-predicted and SVR-predicted EMI for the lag of 18 months without the removal of short-term fluctuations.

Figure S9: The comparison of observed EMI with the RF-predicted and SVR-predicted EMI for the lag of 18 months after removing short-term fluctuations (5-month moving average).

Figure S10: The comparison of observed EMI with the RF-predicted and SVR-predicted EMI for the lag of 24 months without the removal of short-term fluctuations.

Figure S11: The comparison of observed EMI with the RF-predicted and SVR-predicted EMI for the lag of 24 months after removing short-term fluctuations (5-month moving average).

**Table S1:** Latitude/longitude limits of the input variables along with the monthly variance for 6 months lag.

| **Lead**  **Time (months)** | **Input Variable** | **Input Field** | **Latitude** | | **Longitude** | | **Monthly Variance** |
| --- | --- | --- | --- | --- | --- | --- | --- |
| 6 | Sea Surface Temperature Anomaly  (SSTA) | SST1 | -12.25 | 23.90 | -112.15 | 157.22 | 0.2722 |
|  |  | SST2 | 35.86 | 50.88 | -153.53 | -127.34 | 0.1545 |
|  |  | SST3 | -64.92 | -42.99 | -125.77 | 144.13 | 0.4259 |
|  |  | SST4 | -52.84 | -11.22 | 82.31 | 122.13 | 0.0505 |
|  |  | SST5 | -7.06 | 20.41 | -81.24 | -11.04 | 0.0875 |
|  |  | SST6 | 43.86 | 57.35 | -50.33 | -19.95 | 0.0905 |
|  | Sea Surface Height Anomaly  (SSHA) | SSH1 | -11.04 | 7.41 | -95.01 | 165.14 | 0.3784 |
|  |  | SSH2 | 33.86 | 51.14 | -129.59 | 159.79 | 0.0014 |
|  |  | SSH3 | -69.59 | -49.84 | -156.86 | 130.56 | 0.0005 |
|  |  | SSH4 | -66.47 | -40.41 | 60.42 | 91.11 | 0.0021 |
|  |  | SSH5 | 25.39 | 40.61 | -68.70 | -29.74 | 0.0012 |
|  |  | SSH6 | -26.98 | -13.91 | -120.33 | -74.55 | 0.0010 |
|  |  | SSH7 | 1.10 | 22.16 | 110.10 | 162.22 | 0.0003 |
|  |  | SSH8 | -8.14 | 3.04 | 39.97 | 90.13 | 0.0030 |
|  |  | SSH9 | -53.50 | -41.23 | 7.33 | 37.04 | 0.0007 |
|  | Soil Moisture Content Anomaly  (SMC) | SMC1 | 47.88 | 55.90 | 57.04 | 84.82 | 0.0010 |
|  |  | SMC2 | -12.37 | -3.65 | -68.03 | -46.09 | 0.0001 |

|  |  |  |  | |  |  |  |  |  |  |  |  |  |  |  |  |
| --- | --- | --- | --- | --- | --- | --- | --- | --- | --- | --- | --- | --- | --- | --- | --- | --- |
|  | | | |  | | | | | |  |  |  |  |  |  |  |

**Table S2:** Latitude/longitude limits of the input variables along with the monthly variance for 12 months lag.

| **Lead** | **Input Variable** | **Input Field** | **Latitude** | | **Longitude** | | **Monthly Variance** |
| --- | --- | --- | --- | --- | --- | --- | --- |
| 12 | Sea Surface Temperature Anomaly  (SSTA) | SST1 | -5.50 | 9.14 | -166.11 | 151.99 | 0.203861 |
|  |  | SST2 | 35.40 | 52.84 | -171.87 | -130.48 | 0.277876 |
|  |  | SST3 | -66.82 | -48.05 | -158.77 | 144.13 | 0.070235 |
|  |  | SST4 | -36.77 | -13.28 | 93.84 | 121.08 | 0.153831 |
|  |  | SST5 | 9.66 | 24.40 | -72.86 | -17.85 | 0.147339 |
|  |  | SST6 | 47.23 | 56.99 | -47.19 | -23.09 | 0.457068 |
|  |  | SST7 | 0.79 | 7.58 | 68.69 | 84.41 | 0.106139 |
|  |  | SST8 | -12.25 | -4.97 | 39.88 | 61.88 | 0.130835 |
|  | Sea Surface Height Anomaly  (SSHA) | SSH1 | -11.04 | 7.90 | -134.95 | 156.86 | 0.001231 |
|  |  | SSH2 | 35.15 | 55.44 | -162.22 | -133.00 | 0.00045 |
|  |  | SSH3 | -68.48 | -57.67 | -162.71 | 151.99 | 0.001564 |
|  |  | SSH4 | -66.18 | -38.33 | 58.47 | 86.73 | 0.000998 |
|  |  | SSH5 | 24.01 | 40.61 | -64.81 | -45.32 | 0.001487 |
|  |  | SSH6 | -30.12 | -4.75 | -123.26 | -72.60 | 0.000546 |
|  |  | SSH7 | -11.04 | 5.47 | 40.94 | 85.26 | 0.000915 |
|  |  | SSH8 | -53.85 | -41.64 | 7.82 | 51.17 | 1.28E-03 |
|  | Soil Moisture Content Anomaly  (SMC) | SMC1 | 47.88 | 55.90 | 57.04 | 84.82 | 7.24E-05 |
|  |  | SMC2 | -12.37 | -3.65 | -68.03 | -46.09 | 4.61E-05 |

|  |  |  |  |  |  |  |  |  |
| --- | --- | --- | --- | --- | --- | --- | --- | --- |

**Table S3:** Latitude/longitude limits of the input variables along with the monthly variance for 18 months lag.

| **Lag** | **Input Variable** | **Input Field** | **Latitude** | | **Longitude** | | **Monthly Variance** |
| --- | --- | --- | --- | --- | --- | --- | --- |
| 18 | Sea Surface Temperature Anomaly  (SSTA) | SST1 | 34.47 | 45.98 | -160.87 | -134.67 | 0.50045 |
|  |  | SST2 | -59.15 | -38.58 | 119.51 | 170.32 | 0.07904 |
|  |  | SST3 | 48.05 | 56.99 | -50.33 | -21.52 | 0.45003 |
|  |  | SST4 | -2.36 | 22.41 | -87.00 | -30.43 | 0.09916 |
|  |  | SST5 | -25.38 | -3.93 | -135.20 | -71.29 | 0.20747 |
|  |  | SST6 | 1.31 | 17.88 | 52.45 | 85.98 | 0.08641 |
|  |  | SST7 | -19.91 | -3.40 | 41.45 | 63.45 | 0.11162 |
|  | Sea Surface Height Anomaly  (SSHA) | SSH1 | 0.61 | 6.45 | -175.37 | 158.33 | 0.00263 |
|  |  | SSH2 | 30.34 | 46.99 | -135.92 | 160.27 | 0.00057 |
|  |  | SSH3 | -47.18 | -27.88 | 66.75 | 151.99 | 0.00050 |
|  |  | SSH4 | -62.54 | -38.33 | 56.04 | 89.65 | 0.00113 |
|  |  | SSH5 | -50.95 | -31.89 | -150.04 | -91.60 | 0.00046 |
|  |  | SSH6 | -61.91 | -45.63 | -157.35 | -115.46 | 0.00390 |
|  |  | SSH7 | -18.66 | 3.53 | 41.43 | 78.44 | 0.00102 |
|  |  | SSH8 | -56.99 | -42.85 | 10.25 | 46.30 | 0.00116 |
|  | Soil Moisture Content Anomaly  (SMC) | SMC1 | 48.27 | 55.55 | 44.37 | 85.31 | 0.00007 |
|  |  | SMC2 | -12.85 | -3.65 | -68.03 | -42.19 | 0.00005 |

|  |  |  |  |  |  |  |
| --- | --- | --- | --- | --- | --- | --- |

**Table S4:** Latitude/longitude limits of the input variables along with the monthly variance for 24 months lag.

| **Lag** | **Input Variable** | **Input Field** | **Latitude** | | **Longitude** | | **Monthly Variance** |
| --- | --- | --- | --- | --- | --- | --- | --- |
| 24 | Sea Surface Temperature Anomaly  (SSTA) | SST1 | 31.18 | 42.13 | -147.25 | 172.42 | 0.38901 |
|  |  | SST2 | -65.56 | -39.03 | 88.07 | 162.99 | 0.03234 |
|  |  | SST3 | 49.27 | 59.86 | -50.86 | -26.24 | 0.44257 |
|  |  | SST4 | 1.31 | 22.91 | -73.38 | -17.85 | 0.12242 |
|  |  | SST5 | -17.88 | 9.66 | -142.53 | -80.19 | 0.39110 |
|  |  | SST6 | 44.28 | 58.44 | -167.68 | 148.84 | 0.19300 |
|  |  | SST7 | -17.88 | -7.58 | 46.17 | 62.41 | 0.14250 |
|  | Sea Surface Height Anomaly  (SSHA) | SSH1 | 5.96 | 12.72 | -170.50 | 148.58 | 0.00372 |
|  |  | SSH2 | 26.75 | 45.43 | -133.97 | 141.28 | 0.00042 |
|  |  | SSH3 | -47.57 | -27.88 | 45.32 | 158.33 | 0.00043 |
|  |  | SSH4 | -52.78 | -29.23 | -151.51 | -86.24 | 0.00037 |
|  |  | SSH5 | -64.39 | -43.65 | -165.63 | -116.44 | 0.00345 |
|  |  | SSH6 | -21.47 | -10.07 | 51.17 | 85.75 | 0.00153 |
|  |  | SSH7 | -57.33 | -43.65 | 7.82 | 48.73 | 0.00115 |
|  | Soil Moisture Content Anomaly  (SMC) | SMC1 | 41.56 | 54.15 | 47.29 | 85.31 | 0.00004 |
|  |  | SMC2 | -11.89 | -4.63 | -67.05 | -59.25 | 0.00008 |

|  |  |  |  |  |  |  |  |  |
| --- | --- | --- | --- | --- | --- | --- | --- | --- |

Table S5: The model performance metrics for the SVR and RF at four folds and at 6, 12, 18 and 24 months lead time without the removal of short-term fluctuations.

| Support Vector Regression | | | | | | | | | | | | | | | | | |
| --- | --- | --- | --- | --- | --- | --- | --- | --- | --- | --- | --- | --- | --- | --- | --- | --- | --- |
| Metric |  | Lag 6 | | | | Lag 12 | | | | Lag 18 | | | | Lag 24 | | | |
|  |  | Fold | | | | Fold | | | | Fold | | | | Fold | | | |
|  |  | 1 | 2 | 3 | 4 | 1 | 2 | 3 | 4 | 1 | 2 | 3 | 4 | 1 | 2 | 3 | 4 |
| CC | Dev | 0.854 | 0.891 | 0.890 | 0.850 | 0.898 | 0.839 | 0.856 | 0.700 | 0.896 | 0.750 | 0.645 | 0.667 | 0.746 | 0.876 | 0.726 | 0.918 |
|  | Test | 0.633 | 0.692 | 0.870 | 0.680 | 0.542 | 0.683 | 0.723 | 0.555 | 0.306 | 0.358 | 0.493 | 0.468 | 0.551 | 0.702 | 0.671 | 0.466 |
| Dr | Dev | 0.734 | 0.797 | 0.766 | 0.733 | 0.779 | 0.741 | 0.794 | 0.672 | 0.835 | 0.701 | 0.654 | 0.656 | 0.701 | 0.811 | 0.679 | 0.827 |
|  | Test | 0.583 | 0.650 | 0.744 | 0.610 | 0.593 | 0.630 | 0.505 | 0.555 | 0.551 | 0.525 | 0.553 | 0.487 | 0.601 | 0.649 | 0.635 | 0.516 |
| RMSE | Dev | 0.285 | 0.238 | 0.227 | 0.285 | 0.243 | 0.285 | 0.257 | 0.388 | 0.248 | 0.354 | 0.375 | 0.402 | 0.350 | 0.254 | 0.323 | 0.208 |
|  | Test | 0.376 | 0.396 | 0.331 | 0.382 | 0.397 | 0.399 | 0.645 | 0.415 | 0.442 | 0.517 | 0.619 | 0.451 | 0.383 | 0.348 | 0.463 | 0.449 |
| uRMSE | Dev | 0.283 | 0.237 | 0.227 | 0.281 | 0.243 | 0.285 | 0.257 | 0.387 | 0.247 | 0.351 | 0.374 | 0.399 | 0.348 | 0.254 | 0.322 | 0.208 |
|  | Test | 0.372 | 0.382 | 0.298 | 0.359 | 0.395 | 0.396 | 0.440 | 0.404 | 0.442 | 0.502 | 0.517 | 0.420 | 0.381 | 0.335 | 0.458 | 0.428 |
| Random Forest | | | | | | | | | | | | | | | | | |
| Metric |  | Lag 6 | | | | Lag 12 | | | | Lag 18 | | | | Lag 24 | | | |
|  |  | Fold | | | | Fold | | | | Fold | | | | Fold | | | |
|  |  | 1 | 2 | 3 | 4 | 1 | 2 | 3 | 4 | 1 | 2 | 3 | 4 | 1 | 2 | 3 | 4 |
| CC | Dev | 0.926 | 0.927 | 0.987 | 0.915 | 0.881 | 0.845 | 0.813 | 0.794 | 0.957 | 0.943 | 0.847 | 0.881 | 0.881 | 0.910 | 0.879 | 0.869 |
|  | Test | 0.607 | 0.711 | 0.814 | 0.640 | 0.421 | 0.436 | 0.456 | 0.390 | 0.309 | 0.592 | 0.319 | 0.390 | 0.512 | 0.559 | 0.434 | 0.307 |
| Dr | Dev | 0.796 | 0.800 | 0.905 | 0.787 | 0.759 | 0.728 | 0.686 | 0.679 | 0.813 | 0.807 | 0.718 | 0.736 | 0.738 | 0.770 | 0.739 | 0.719 |
|  | Test | 0.605 | 0.633 | 0.702 | 0.591 | 0.528 | 0.591 | 0.567 | 0.449 | 0.534 | 0.610 | 0.551 | 0.478 | 0.561 | 0.572 | 0.534 | 0.513 |
| RMSE | Dev | 0.231 | 0.214 | 0.101 | 0.237 | 0.283 | 0.304 | 0.324 | 0.371 | 0.214 | 0.214 | 0.293 | 0.299 | 0.289 | 0.253 | 0.259 | 0.306 |
|  | Test | 0.368 | 0.388 | 0.389 | 0.396 | 0.461 | 0.518 | 0.573 | 0.493 | 0.437 | 0.443 | 0.575 | 0.470 | 0.411 | 0.399 | 0.574 | 0.461 |
| uRMSE | Dev | 0.231 | 0.214 | 0.101 | 0.237 | 0.282 | 0.304 | 0.324 | 0.371 | 0.214 | 0.214 | 0.293 | 0.299 | 0.289 | 0.253 | 0.259 | 0.306 |
|  | Test | 0.359 | 0.388 | 0.364 | 0.383 | 0.409 | 0.488 | 0.545 | 0.441 | 0.432 | 0.443 | 0.561 | 0.440 | 0.400 | 0.386 | 0.543 | 0.454 |

Table S6: The model performance metrics for the SVR and RF at four folds and at 6, 12, 18 and 24 months lead time after removing the short-term fluctuations.

| Support Vector Regression | | | | | | | | | | | | | | | | | |
| --- | --- | --- | --- | --- | --- | --- | --- | --- | --- | --- | --- | --- | --- | --- | --- | --- | --- |
| Metric |  | Lag 6 | | | | Lag 12 | | | | Lag 18 | | | | Lag 24 | | | |
|  |  | Fold | | | | Fold | | | | Fold | | | | Fold | | | |
|  |  | 1 | 2 | 3 | 4 | 1 | 2 | 3 | 4 | 1 | 2 | 3 | 4 | 1 | 2 | 3 | 4 |
| CC | Dev | 0.910 | 0.946 | 0.952 | 0.912 | 0.946 | 0.905 | 0.907 | 0.761 | 0.951 | 0.854 | 0.709 | 0.741 | 0.823 | 0.932 | 0.796 | 0.965 |
|  | Test | 0.735 | 0.773 | 0.910 | 0.812 | 0.626 | 0.751 | 0.801 | 0.706 | 0.429 | 0.368 | 0.573 | 0.544 | 0.615 | 0.795 | 0.743 | 0.566 |
| Dr | Dev | 0.786 | 0.847 | 0.843 | 0.786 | 0.839 | 0.801 | 0.821 | 0.710 | 0.864 | 0.738 | 0.682 | 0.691 | 0.740 | 0.836 | 0.717 | 0.873 |
|  | Test | 0.654 | 0.684 | 0.778 | 0.662 | 0.631 | 0.670 | 0.517 | 0.578 | 0.597 | 0.533 | 0.582 | 0.504 | 0.613 | 0.698 | 0.664 | 0.548 |
| RMSE | Dev | 0.218 | 0.165 | 0.150 | 0.217 | 0.176 | 0.218 | 0.197 | 0.338 | 0.184 | 0.286 | 0.328 | 0.359 | 0.290 | 0.188 | 0.269 | 0.142 |
|  | Test | 0.299 | 0.332 | 0.273 | 0.309 | 0.335 | 0.343 | 0.610 | 0.354 | 0.375 | 0.495 | 0.583 | 0.397 | 0.333 | 0.293 | 0.421 | 0.382 |
| uRMSE | Dev | 0.216 | 0.164 | 0.150 | 0.210 | 0.176 | 0.218 | 0.197 | 0.337 | 0.181 | 0.283 | 0.325 | 0.354 | 0.287 | 0.187 | 0.268 | 0.142 |
|  | Test | 0.294 | 0.316 | 0.237 | 0.280 | 0.332 | 0.340 | 0.400 | 0.342 | 0.375 | 0.485 | 0.479 | 0.360 | 0.331 | 0.275 | 0.416 | 0.358 |
| Random Forest | | | | | | | | | | | | | | | | | |
| Metric |  | Lag 6 | | | | Lag 12 | | | | Lag 18 | | | | Lag 24 | | | |
|  |  | Fold | | | | Fold | | | | Fold | | | | Fold | | | |
|  |  | 1 | 2 | 3 | 4 | 1 | 2 | 3 | 4 | 1 | 2 | 3 | 4 | 1 | 2 | 3 | 4 |
| CC | Dev | 0.953 | 0.956 | 0.990 | 0.947 | 0.927 | 0.892 | 0.879 | 0.841 | 0.974 | 0.966 | 0.891 | 0.920 | 0.937 | 0.956 | 0.915 | 0.925 |
|  | Test | 0.683 | 0.802 | 0.874 | 0.794 | 0.600 | 0.476 | 0.528 | 0.543 | 0.403 | 0.623 | 0.413 | 0.537 | 0.615 | 0.679 | 0.528 | 0.413 |
| Dr | Dev | 0.827 | 0.835 | 0.921 | 0.820 | 0.799 | 0.760 | 0.718 | 0.700 | 0.837 | 0.835 | 0.746 | 0.766 | 0.769 | 0.799 | 0.768 | 0.746 |
|  | Test | 0.633 | 0.671 | 0.734 | 0.631 | 0.542 | 0.603 | 0.585 | 0.453 | 0.557 | 0.631 | 0.576 | 0.478 | 0.556 | 0.615 | 0.550 | 0.535 |
| RMSE | Dev | 0.185 | 0.167 | 0.085 | 0.190 | 0.230 | 0.256 | 0.274 | 0.331 | 0.182 | 0.173 | 0.251 | 0.261 | 0.241 | 0.200 | 0.220 | 0.264 |
|  | Test | 0.317 | 0.338 | 0.334 | 0.324 | 0.401 | 0.483 | 0.537 | 0.445 | 0.384 | 0.434 | 0.539 | 0.415 | 0.367 | 0.355 | 0.540 | 0.400 |
| uRMSE | Dev | 0.185 | 0.167 | 0.085 | 0.190 | 0.230 | 0.256 | 0.274 | 0.331 | 0.182 | 0.173 | 0.251 | 0.261 | 0.241 | 0.200 | 0.220 | 0.264 |
|  | Test | 0.308 | 0.338 | 0.309 | 0.308 | 0.343 | 0.447 | 0.510 | 0.387 | 0.380 | 0.432 | 0.527 | 0.380 | 0.355 | 0.343 | 0.510 | 0.393 |


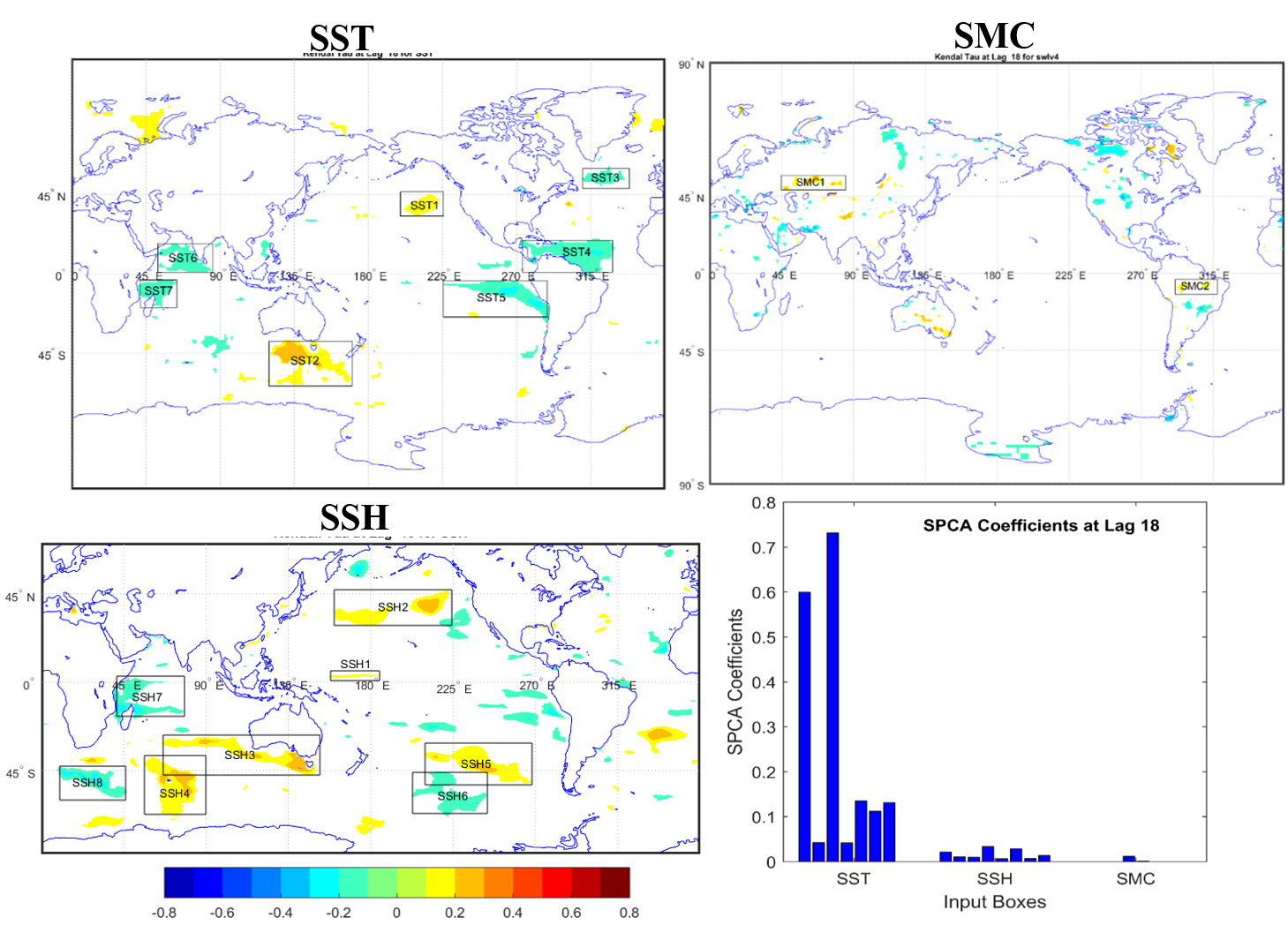


Figure S1: Identified significant zones from the global anomaly fields of SSTA, SSHA and SMC along with the SPCA coefficients of each identified zones at 18 months lag.


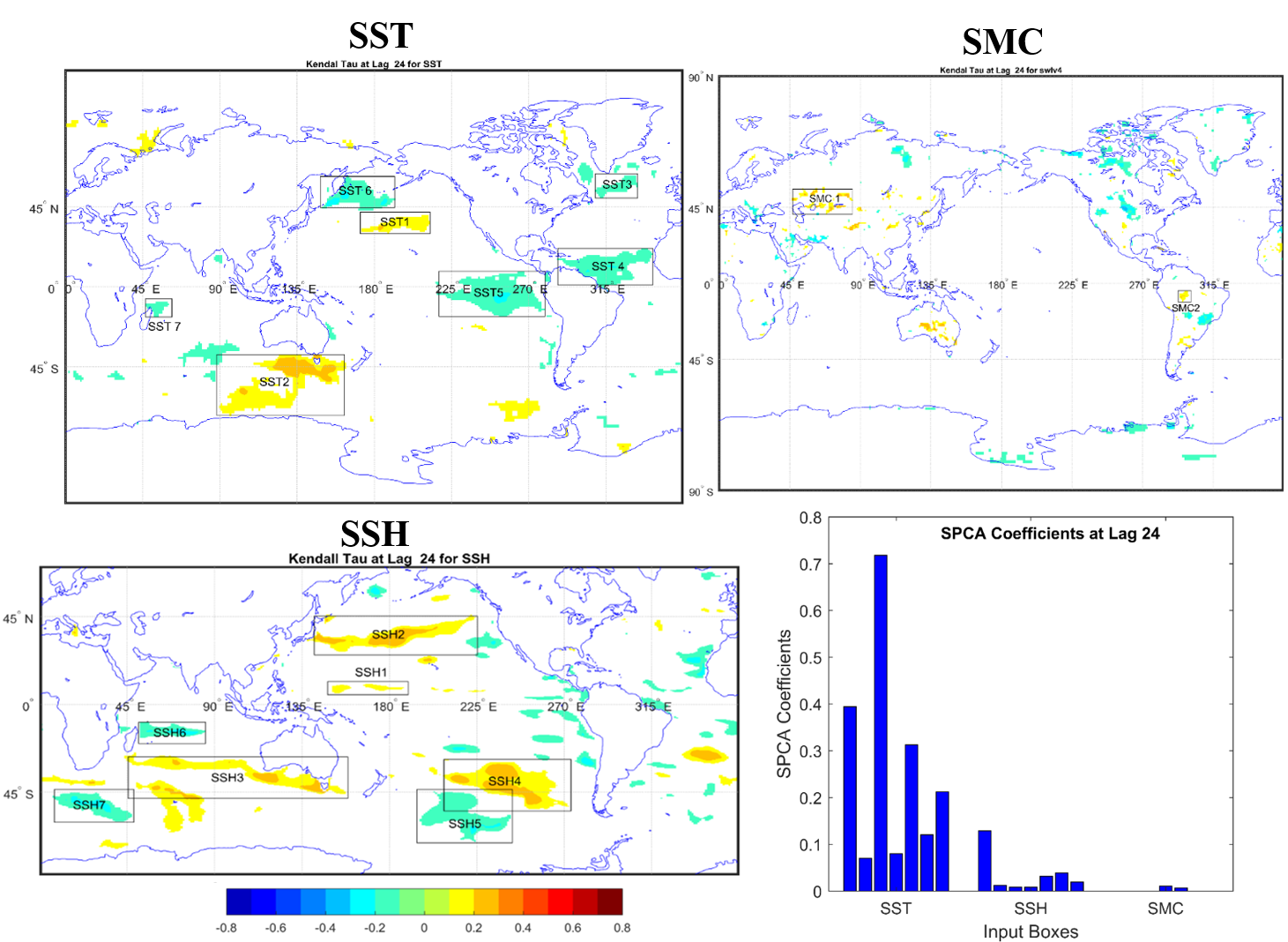


Figure S2: Same as Figure 1 but at 24 months lag.


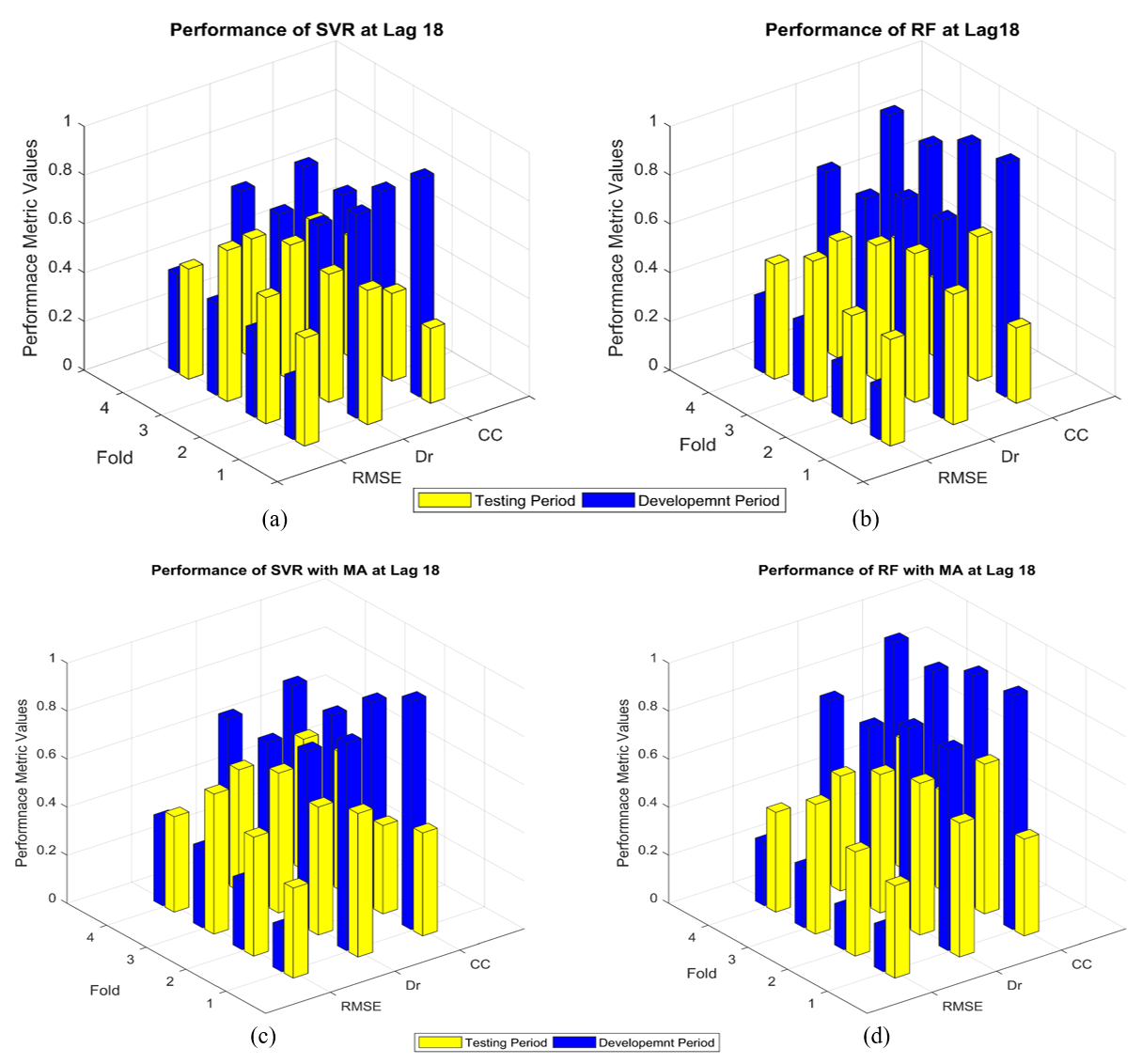


Figure S3: Comparison of model performances with 18 months lead time: (a) Performance of SVR without applying the MA; (b) Performance of RF without applying the MA; (c) Performance of SVR after applying the MA; and (d) Performance of RF after applying the MA.


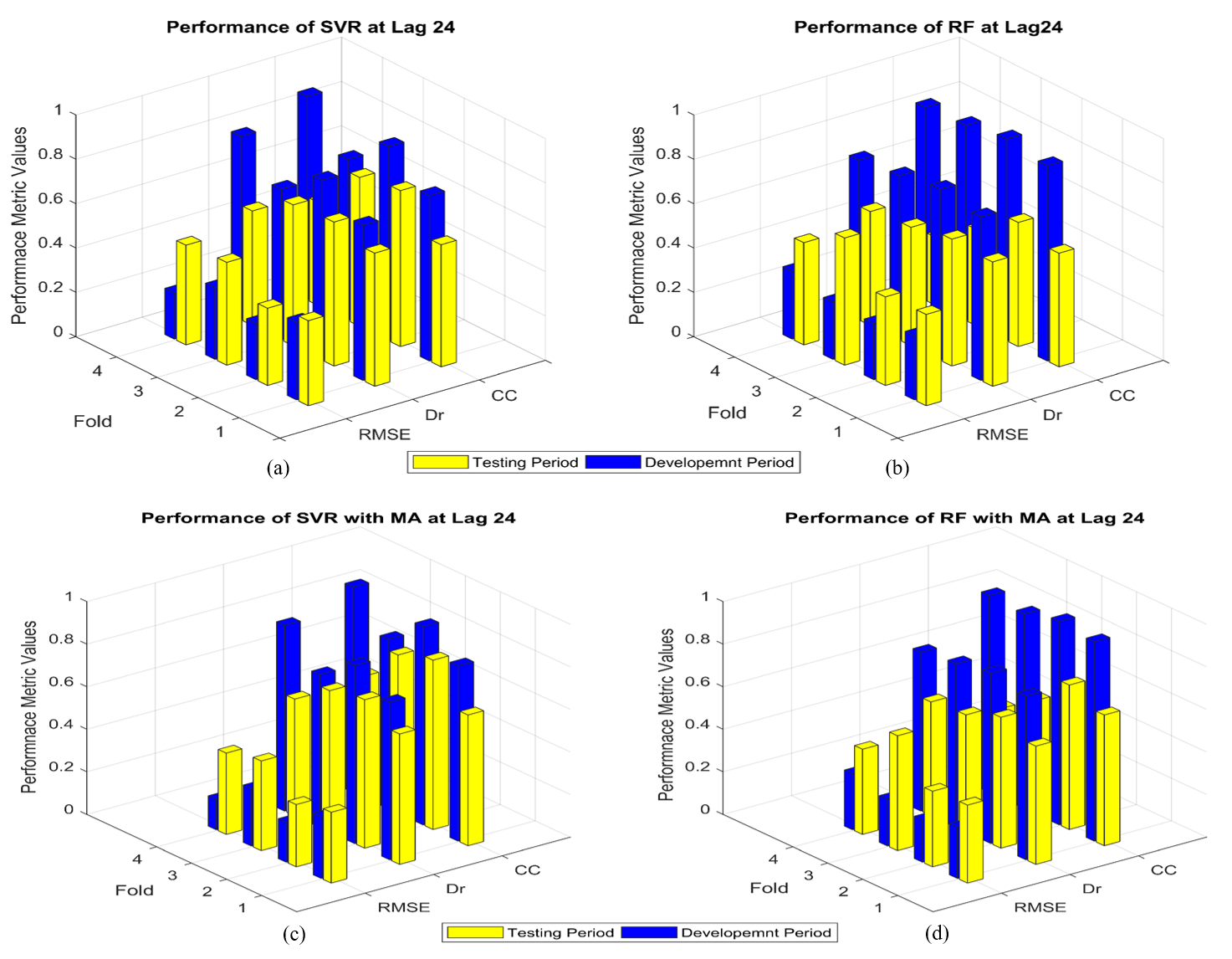


Figure S4: Comparison of model performances with 24 months lead time: (a) Performance of SVR without applying the MA; (b) Performance of RF without applying the MA; (c) Performance of SVR after applying the MA; and (d) Performance of RF after applying the MA.


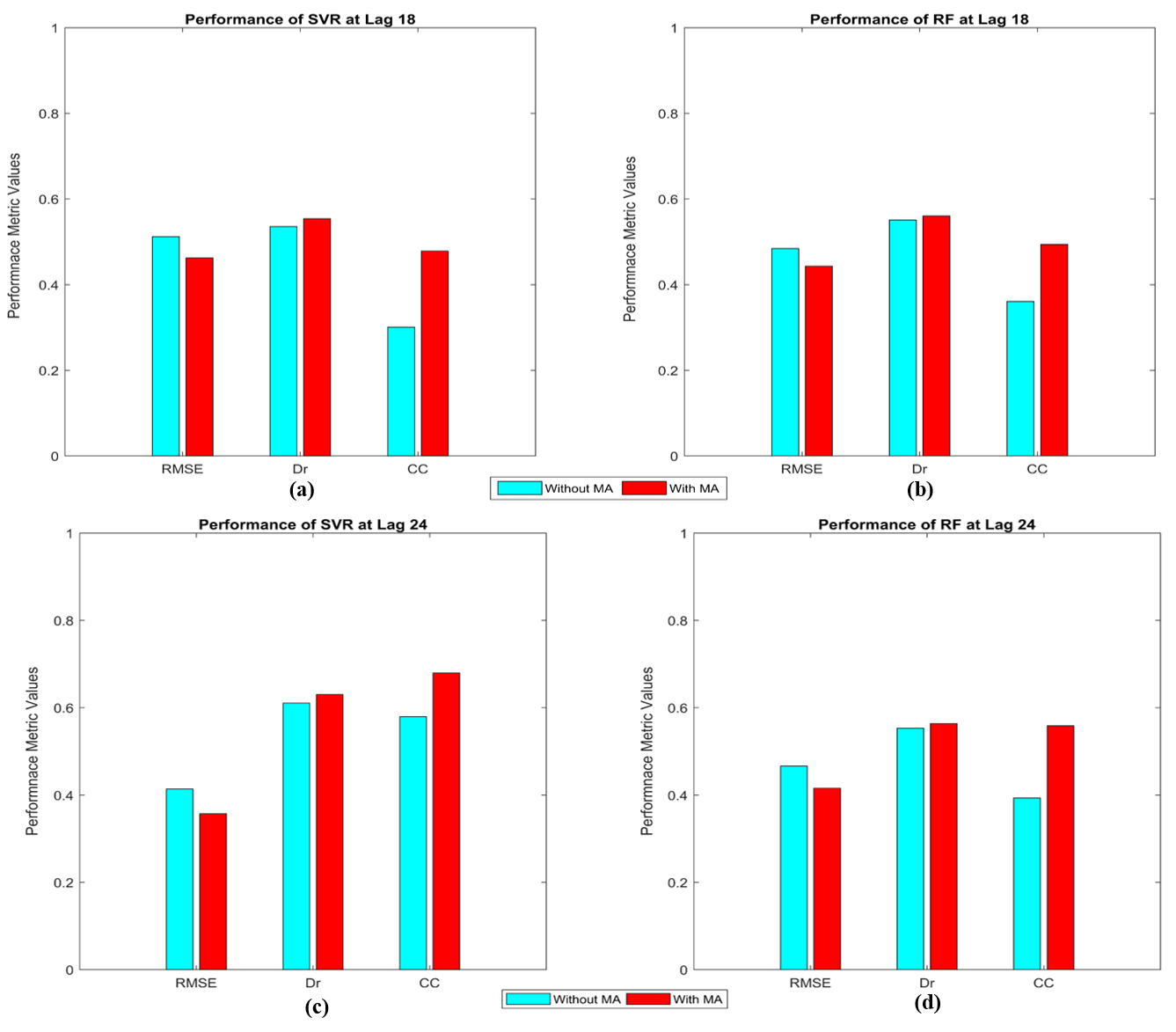


Figure S5: Comparison of overall model performances without and with applying the MA to remove short-term fluctuations: (a) Performance of SVR with lead time of 18 months; (b) Performance of RF with lead time of 18 months; (c) Performance of SVR with lead time of 24 months; and (d) Performance of RF with lead time of 24 months.


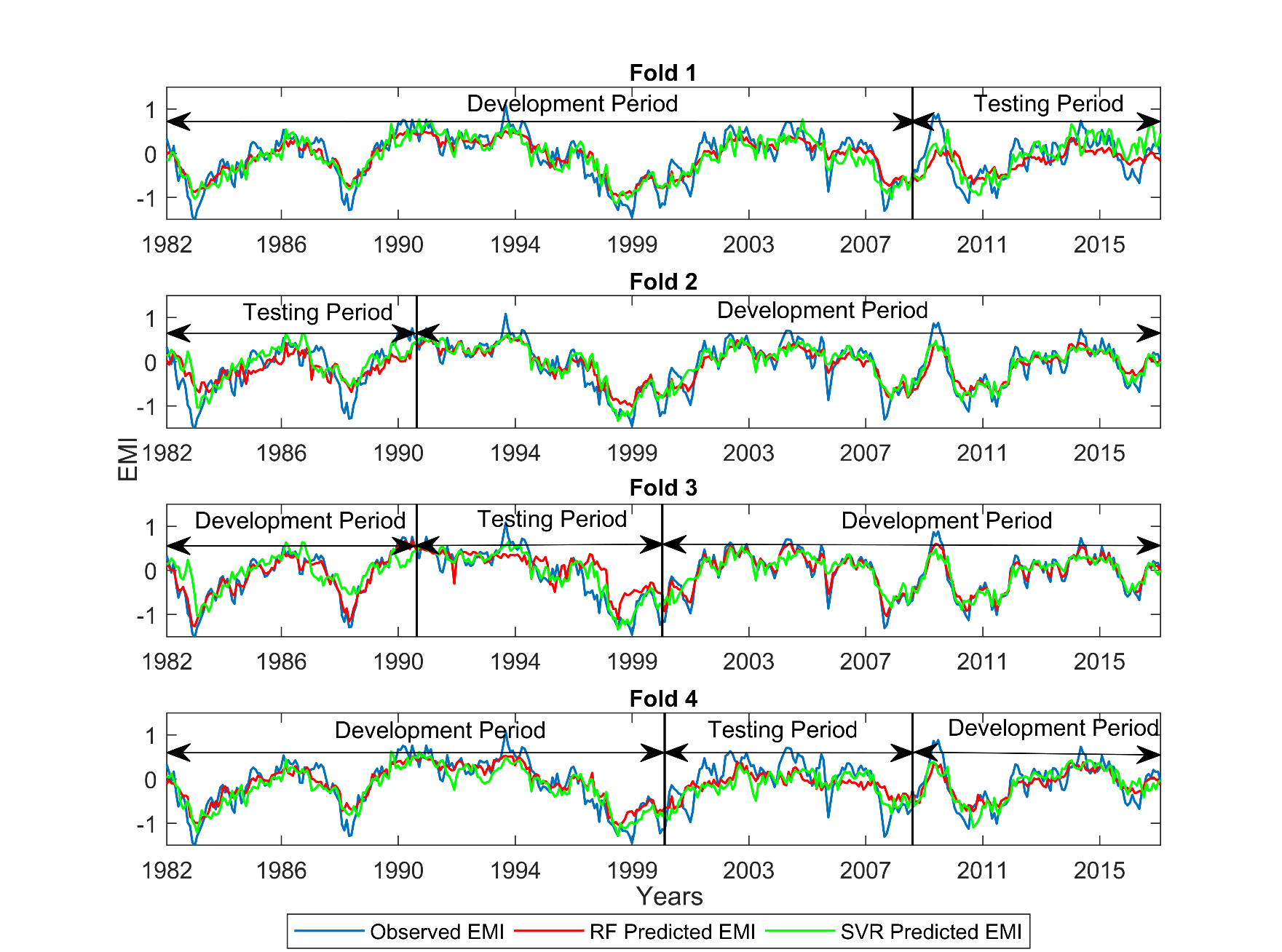


Figure S6: Comparison of observed EMI with the RF-predicted and SVR-predicted EMI with 6 months lead time without the removal of short-term fluctuations.


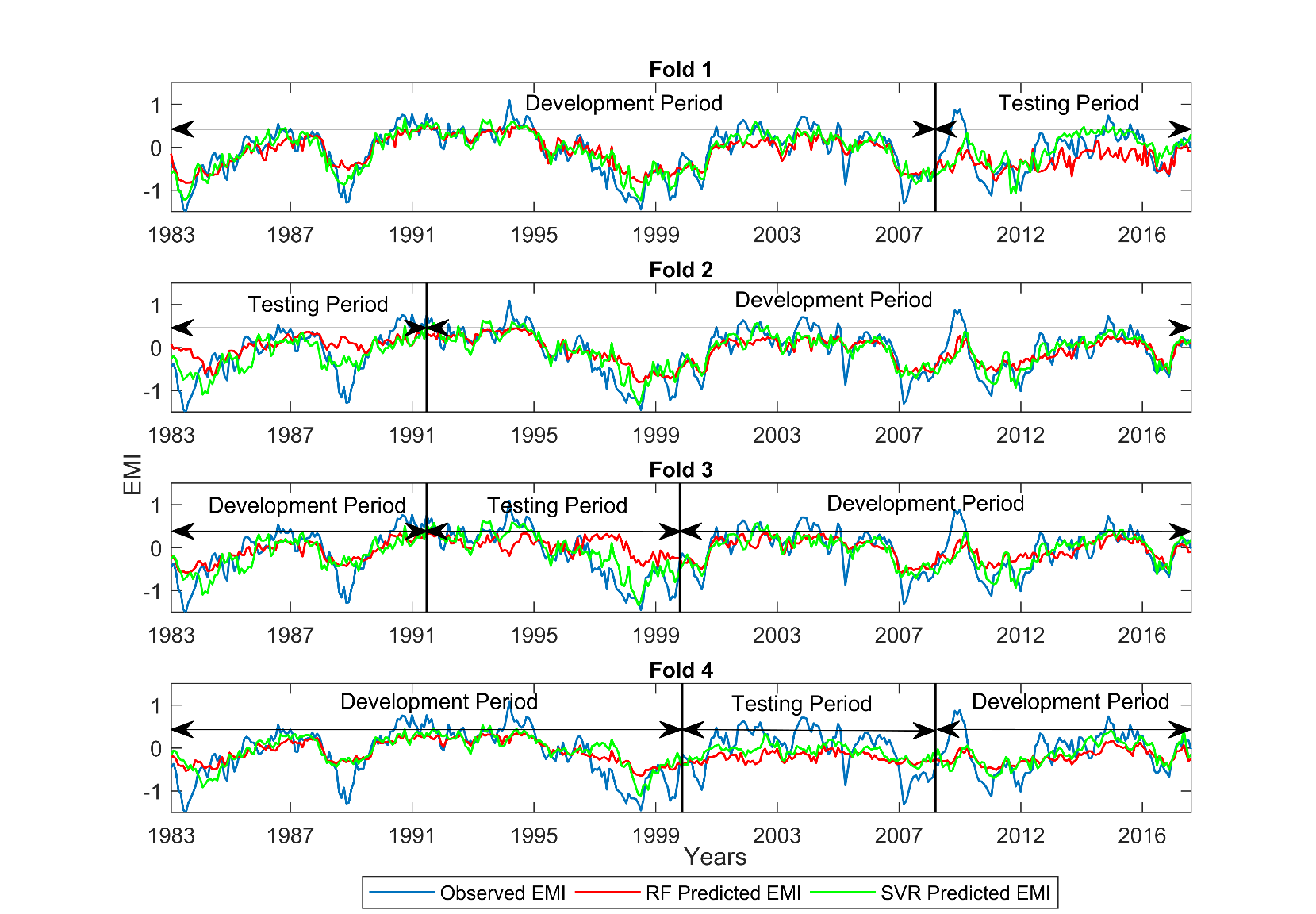
Figure S7: Comparison of observed EMI with the RF-predicted and SVR-predicted EMI at 12 months lead time without the removal of short-term fluctuations.


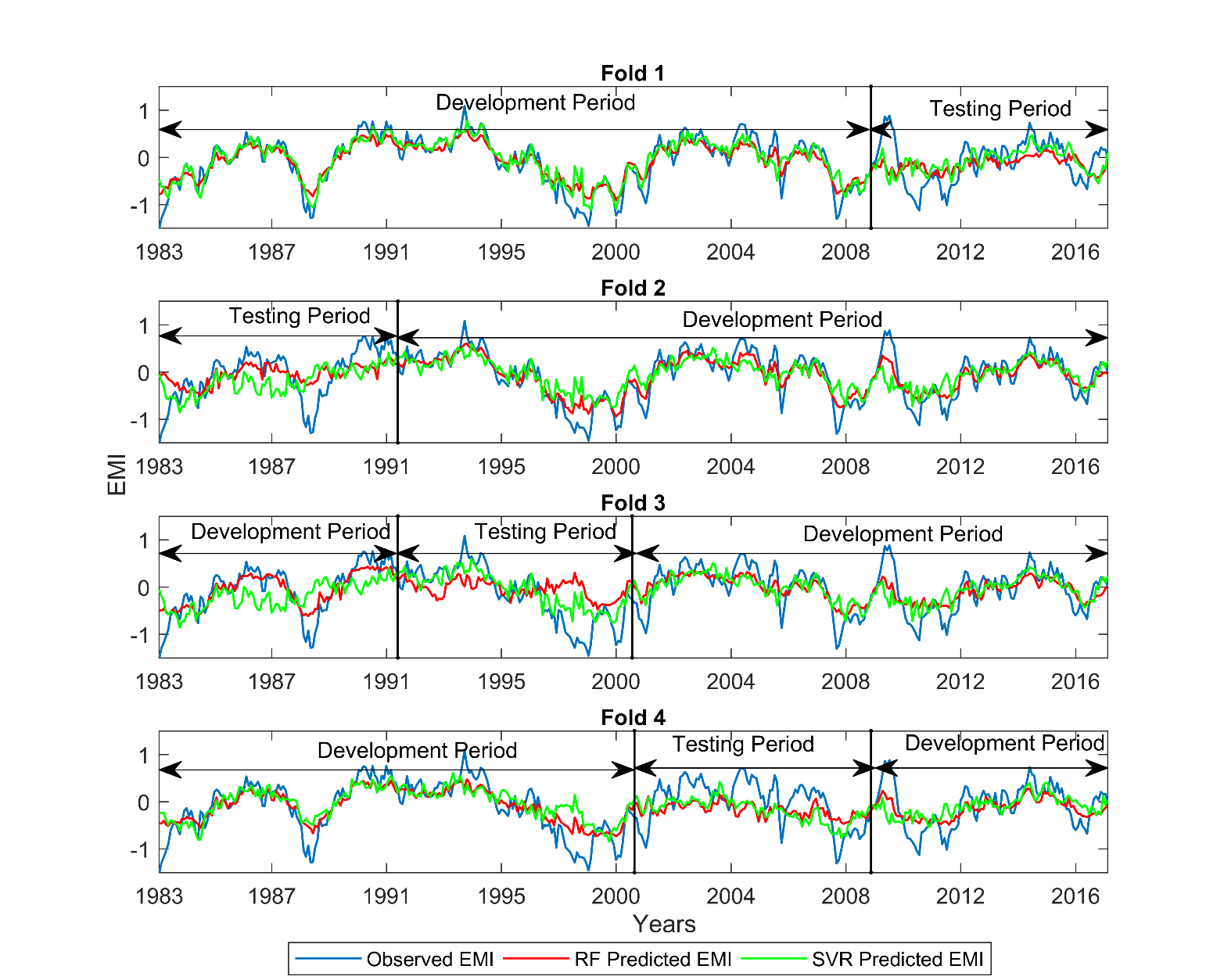


Figure S8: The comparison of observed EMI with the RF-predicted and SVR-predicted EMI for the lag of 18 months without the removal of short-term fluctuations.


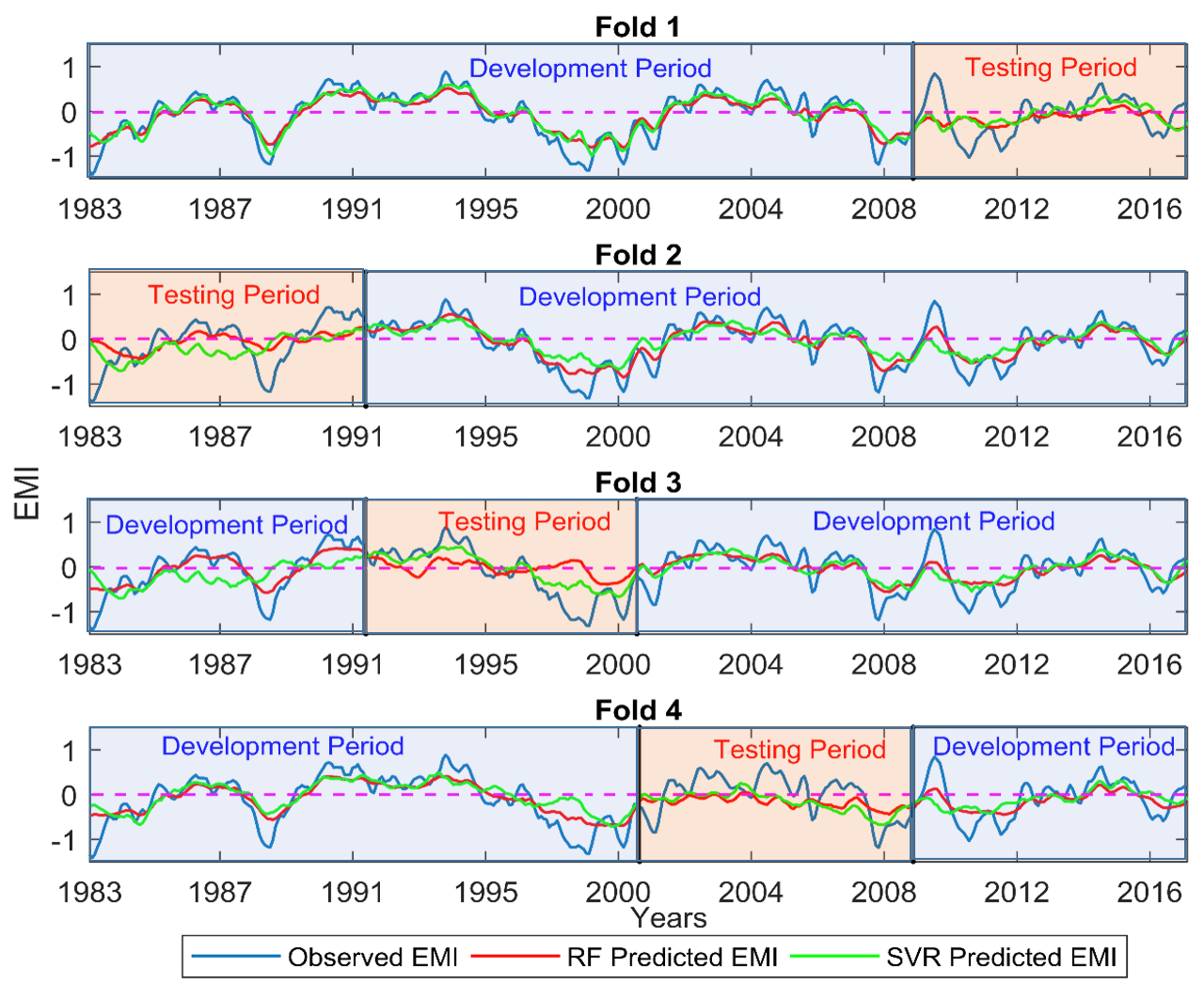


Figure S9: The comparison of observed EMI with the RF-predicted and SVR-predicted EMI for the lag of 18 months after removing short-term fluctuations (5-month moving average).


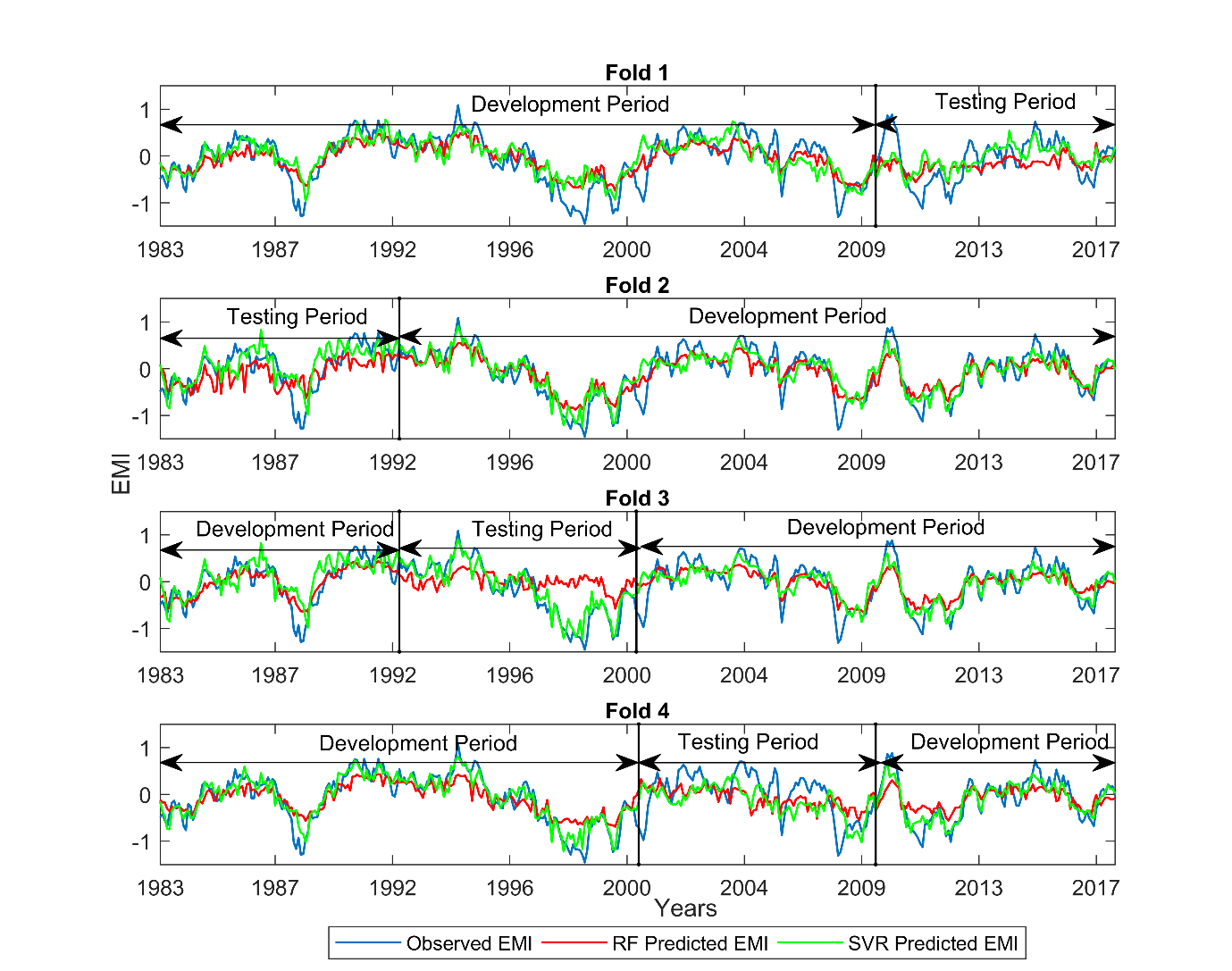


Figure S10: The comparison of observed EMI with the RF-predicted and SVR-predicted EMI for the lag of 24 months without the removal of short-term fluctuations.


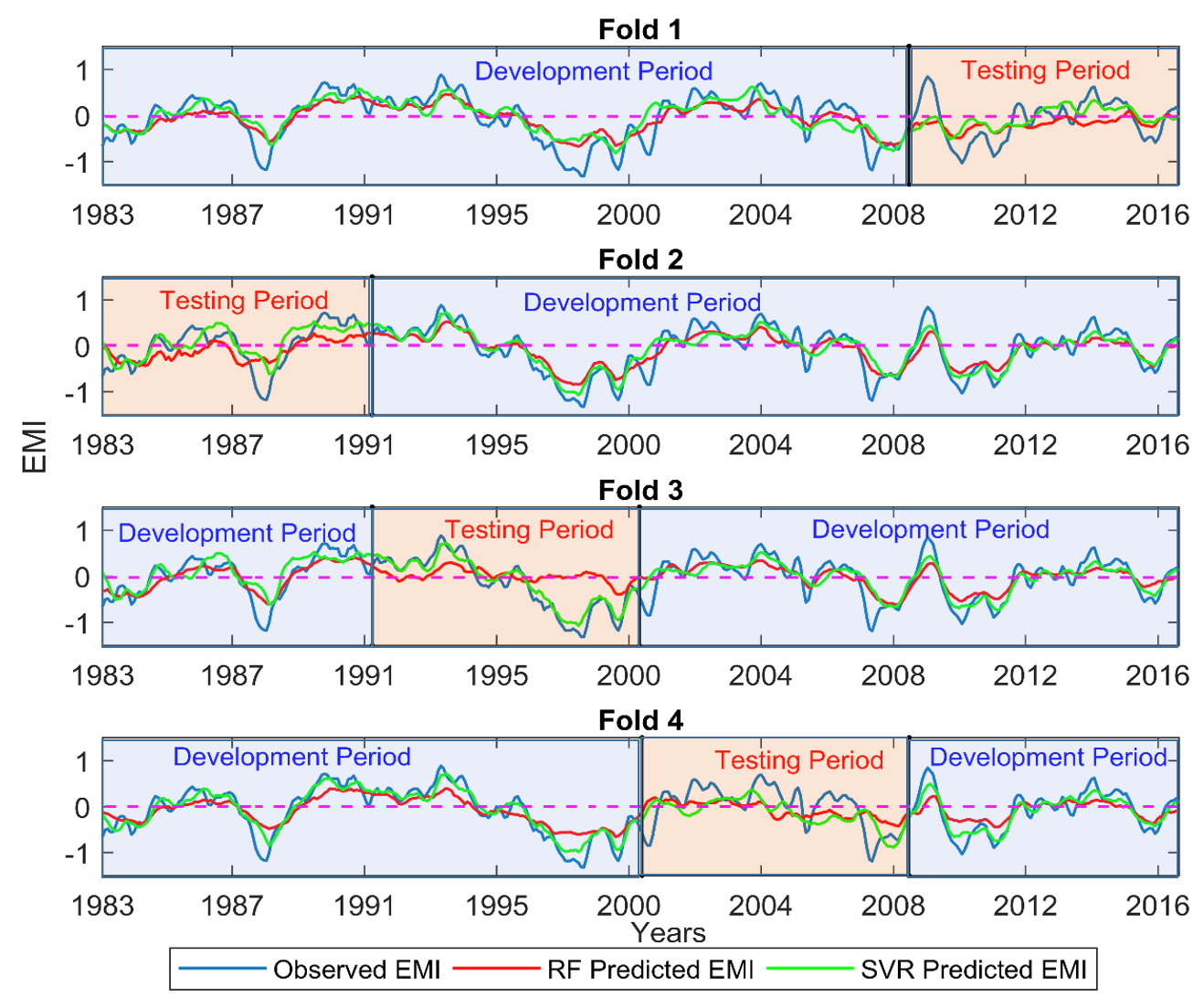


Figure S11: The comparison of observed EMI with the RF-predicted and SVR-predicted EMI for the lag of 24 months after removing short-term fluctuations (5-month moving average).

1. Corresponding Author: Dr. Rajib Maity, email: [rajib@civil.iitkgp.ac.in](mailto:rajib@civil.iitkgp.ac.in), [rajibmaity@gmail.com](mailto:rajibmaity@gmail.com). [↑](#footnote-ref-1)
